# MorbiNet: multimorbidity networks in adult general population. Analysis of type 2 diabetes mellitus comorbidity

**DOI:** 10.1038/s41598-020-59336-1

**Published:** 2020-02-12

**Authors:** Alba Aguado, Ferran Moratalla-Navarro, Flora López-Simarro, Victor Moreno

**Affiliations:** 1 CAP Sagrada Familia. Consorci Sanitari Integral, Barcelona, Spain; 2 Oncology Data Analytics Program, Catalan Institute of Oncology (ICO), L’Hospitalet de Llobregat, Barcelona, Spain; 3 ONCOBELL Program, Bellvitge Biomedical Research Institute (IDIBELL), L’Hospitalet de Llobregat, Barcelona, Spain; 4 CIBER of Epidemiology and Public Health (CIBERESP), Madrid, Spain; 5 Department of Clinical Sciences, Faculty of Medicine and Health Sciences, University of Barcelona, Barcelona, Spain; 6 ABS Urban Martorell, Catalan Institute of Health, Martorell, Barcelona, Spain

**Keywords:** Diabetes, Comorbidities

## Abstract

Multimorbidity has great impact on health care. We constructed multimorbidity networks in the general population, extracted subnets focused on common chronic conditions and analysed type 2 diabetes mellitus (T2DM) comorbidity network. We used electronic records from 3,135,948 adult people in Catalonia, Spain (539,909 with T2DM), with at least 2 coexistent chronic conditions within the study period (2006–2017). We constructed networks from odds-ratio estimates adjusted by age and sex and considered connections with OR > 1.2 and p-value < 1e-5. Directed networks and trajectories were derived from temporal associations. Interactive networks are freely available in a website with the option to customize characteristics and subnets. The more connected conditions in T2DM undirected network were: complicated hypertension and atherosclerosis/peripheral vascular disease (degree: 32), cholecystitis/cholelithiasis, retinopathy and peripheral neuritis/neuropathy (degree: 31). T2DM has moderate number of connections and centrality but is associated with conditions with high scores in the multimorbidity network (neuropathy, anaemia and digestive diseases), and severe conditions with poor prognosis. The strongest associations from T2DM directed networks were to retinopathy (OR: 23.8), glomerulonephritis/nephrosis (OR: 3.4), peripheral neuritis/neuropathy (OR: 2.7) and pancreas cancer (OR: 2.4). Temporal associations showed the relevance of retinopathy in the progression to complicated hypertension, cerebrovascular disease, ischemic heart disease and organ failure.

## Introduction

Multimorbidity is the simultaneous presence of two or more chronic medical conditions^1^. It is a common problem, more than one-third of the patients visited by primary care physicians have four or more chronic health problems and a small proportion has more than ten during their life^2^. The prevalence is higher in older people and low socioeconomic situations^3^. It is associated with a poorer quality of life, more disability^4^ and patient safety incidents^5^ and a greater, almost exponential, increase in health care costs^6^.

Multimorbidity has raised increasing interest in the last years. It is a complex phenomenon and can be studied with network analysis. Hidalgo *et al*. studied phenotypic networks and found that patients with diseases highly connected tend to die sooner^7^. This tool has been used to identify comorbidity associated with hypertension^8^, chronic pulmonary obstructive disease^9^ or mental disease^10^ and to compare multimorbidity by gender^11^. Most of the studies used hospital databases to construct the networks. It could be useful to approach the study of multimorbidity for an individual patient focusing on an initial condition or the one with the highest impact. Thus, comorbidity networks could help to analyse multimorbidity.

There are 425 million people with diabetes in the world and the prevalence is increasing (IDF Atlas) because of the aging population and lifestyle habits^12^. In Spain, the adjusted prevalence for age and sex of diabetes mellitus was estimated to be 13.8% (6.0% being undiagnosed)^12^. Comorbidities are common in patients with T2DM^13^ and the proportion of diabetic patients with multimorbidity increases after diagnosis (from 32% to 80% after 16 years)^14^. Multimorbidty in diabetics is associated with a reduced quality of life^15^, increased cost^16^ and mortality^17^. Diseases associated with diabetes have widely been studied, but not using the approach of network analysis.

We aimed to construct the multimorbidity network in the general population, extract subnets focused on the most common chronic health conditions, construct an interactive website openly available to visualize the networks and analyse T2DM comorbidity network.

## Methods

### Study design, population and variables

We performed a retrospective longitudinal population-based study in adults in Catalonia, Spain. We used electronic health records from the Information System for Research Development in Primary Care (SIDIAP), which includes clinical data from approximately 5.5 million people, 74% of the population in Catalonia. All of them are users of the public health care system.

We included subjects aged 18 years or more with at least two coexistent chronic health conditions active any time within the period 2006–2017. Diseases were coded with the International Statistical Classification of Diseases and Related Health Problems 10th revision (ICD-10) system^18^ in the electronic records. ICD-10 code diagnoses were mapped to the International Classification of Primary Care, 2^nd^ edition (ICPC-2) system^19^. We followed an adapted version of the list of chronic conditions based on ICPC-2 codes described by O’Halloran^20^. We obtained the variables: date of birth, sex, diagnosis (date of diagnosis and resolution, ICPC-2 code), tobacco and alcohol consumption, socioeconomic group, weight, height and rurality). Patient’s age was defined as the mean value between the age when the first and last diagnoses were made within the study period or the age in the middle of the period for the cases without new diagnoses made in the period.

For the T2DM comorbidity analysis, we included the patients with multimorbidity with the ICPC-2 code T90 (diabetes non-insulin dependent), originally coded in ICD-10 as E11 (type 2 diabetes mellitus).

### Network construction and analysis

We used logistic regression models, adjusted by age and sex, to construct the multimorbidity networks. A relational database was developed on a server to store the variables at the individual level, with a table for patient characteristics and another one for multiple diagnoses records. We used free R software and R Igraph library to prepare database, construct the networks and obtain network parameters and node attributes. The conditions with less than 1000 patients were not included in the networks. For the patients who died or transferred to a different region, we included the data until the date of death or transfer.

The basic elements of the networks are nodes (chronic health conditions) and edges (coexistence of disease) that connect nodes within the network. Graphs are visual representations of networks, with nodes and edges. Connections are defined according to the criteria of coexistence to a greater degree than expected by the prevalence of diseases. For these criteria, thresholds were used in the association measures (odds ratios (OR) ≥ 1.2) and p-value < 1e-5 (Bonferroni correction to account for multiple tests). These filters we applied because the large sample size analysed would generate significant associations even for very small magnitudes. Edge thickness in the network plots varied proportional to OR values. We constructed non-directional networks, and also directed networks and trajectories for the specific analysis of temporal associations.

### Directed networks and trajectories

We constructed directed networks to assess temporal disease associations. We identified sequential associations among pairs of diagnoses to study temporal patterns. We only considered a temporal association for probabilities below 40% or above 60% that a disease was diagnosed previously or afterward another one. Conditions diagnosed between 41 and 59% of the times before or after another one were considered to have no temporal association. Thus, a diagnosis D_a_ was considered to precede diagnosis D_b_ when in at least 60% of patients it took place before (sequence D_a_ - D_b_). When D_a_ was made before D_b_ in less than 40% of the patients we assumed the sequence was D_b_ - D_a_. This approach might be useful to analyse disease progression and study temporal directionality in multimorbidity.

With the directed networks for a specific disease, we constructed a trajectory. We considered only directed associations with odds ratios ≥ 1.5. The trajectory was made with the conditions connected to the disease of interest (first steps before it) and other chronic diseases connected to (up to three steps from the disease of interest).

### Node attributes and network parameters

We obtained relevant node attributes such as the degree, clustering coefficient and PageRank index. The *degree* (*K*_*i*_) measures the connectivity of the node^21^, as the number of links connected to node *i*. The *clustering coefficient* measures the likelihood that two nodes connected to node *i* are connected themselves. It is a density measure of local connections^22^ and shows the tendency of the network to aggregate in subgroups. It’s a number between 0 and 1, and is calculated as^21^:

$$Clustering\,coefficien{t}_{i}=\frac{2{e}_{i}}{{k}_{i}({k}_{i}-1)}$$

Where *e*_*i*_ is the number of existing links among the *k*_*i*_ nodes that connect to node *i*

The *pageRank* measures node influence, based on the number of links it has to other nodes in the network and the links their connections have, taking into account links direction and weight.

In a directed network PageRank index for disease *g*, *PR(g)*, is calculated as^23^:

$$PR(g)=(1-d){N}^{-1}+{\sum }_{u\in U(g)}PR(u)/{N}_{ds}(u).$$

where *U(g)* is the set of inbound conditions of disease *g*, *N*_*ds*_*(u)* is the number of outbound diseases of disease *u* and *N* is the total number of chronic conditions in the underlying network and *d* is a damping factor that has been set to 0.85.

In an undirected weighted network, the edges are considered as bi-directional and the classic centralities definition is applied.

Regarding the whole network, we calculated the number of nodes and edges, the clustering coefficient, diameter, density and centralization:

The *Number of nodes* is the number of network units and the *Number of edges* the number of links connecting the nodes.

The network *clustering coefficient* is the average of the clustering coefficients for all nodes in the network. It measures the degree to which nodes in a graph tend to cluster together.

*Network diameter* is the maximum distance between any two nodes in a network^21^:

$$Network\,Diameter=max\{{d}_{ij}|i,j{\epsilon }N\}$$

where *N* is the set of nodes in the network.

The *network density* is defined as the number of connections between nodes divided by the number of possible ties or connections^22^. It is a measure of the relative number of connections. It is a value between 0 and 1. A network with only isolated nodes has a density of 0. It is calculated as:

$$Density=\frac{{\sum }_{i}{\sum }_{j}{m}_{ij}}{n(n-1)}$$

where *M* = [*m*_*ij*_] is the adjacency *n*×*n* matrix

*Centralization* is the extent to which network links are focused on one or a few nodes^24^. It is the difference between the number of links for each node divided by the maximum possible sum of differences. It indicates the degree to which a network approaches the configuration of a star network and is related to the degree of asymmetry. Centralization is expressed as a percentage, and it has values from 0 (every node is connected to every other node) to 100 (all nodes are connected to only one node). A centralized network will have many of its links around one or a few nodes.

### Subnets focused on chronic conditions

The most common chronic conditions were zoomed, and subnets extracted from the general multimorbidity network. We analysed here the T2DM subnet with ICPC-2 codes and OR ≥ 1.2.

### Quality of data and sensitivity analyses

Since this study was based on routinely collected data, and that can vary from doctor to doctor, we selected the group of patients who had recorded in their electronic health record the variables tobacco and alcohol consumption, weight and height, as a measure of better-quality records. The subgroup of patients with all these variables correctly recorded was 2,214,388 (70% from total) and was compared to the patients with any of them missing. We studied the correlations for the prevalence of diseases in both groups and only for the more infrequent chronic conditions the differences were slightly lower (for the different diseases r^2^ ranged from 0.94 to 0.98). The prevalence of most chronic conditions was similar in both groups. Because of this, we used the data of the entire population to construct the networks. Patients with a higher burden of disease would be expected to have more clinical information recorded. For health problems where alcohol, tobacco or weight are risk factors this information might be more carefully obtained. Older patients have more morbidity, are visited more often and have more chances to have this data in their health records. If we would have included only the patients with better quality records, we might be selecting the population with more diseases and introducing bias to the study.

As sensitivity analyses, we analysed how T2DM ICPC-2 networks changed when we modified several conditions (threshold OR value, including only the patients with more complete data versus all patients, changing the criteria to define directionality with probabilities <20% or >80% of previous/subsequent diagnosis among pairs of conditions versus <40% or >60%).

### Website

The networks and results of the study can be freely accessed on an open website^25^. We have developed an application where users can visualize interactively different networks according to the selection of characteristics of interest. We used Shiny free software (shiny.rstudio.com), R visNetwork library and R Igraph library to visualize networks and obtained descriptive and topological parameters.

The networks can be viewed with different formats to analyse the relationships among diseases. It is possible to select the disease classification system: ICD-10 with 3 digits, ICPC-2 and a simplified ICPC-2 version listed in Supplementary Table S1. It is also possible to select the minimum odds ratio considered to accept a risk association is present (ranging from 1.2 to 2) and to plot the global multimorbidity network or zoom the subnet of specific chronic conditions. It is also possible to represent protective interactions, with a maximum OR to accept them ranging from 0.8 to 0.5.

The node size shown in plots depends on the degree of the condition in the network and the node colour can be selected according to different criteria: clustering coefficient, pageRank value, system location (such as cardiovascular, respiratory…). For a chronic condition subnet, users can select the colour and the size of the nodes according to the selected attribute value in the general network or within the subnet.

The network parameters can be visualized and downloaded. These include the number of nodes and edges, diameter, shortest pathway, density, average neighbours, clustering coefficient and centralization, both for the general network or subnetwork. The degree for each node and OR for each pair of nodes can also be visualized and downloaded.

Directed networks (or subnetworks) can also be plotted for the selected disease classification system and OR value and it is possible to visualize the network descriptive parameters and node attributes.

The website also includes a tab to obtain descriptive statistics of the study population with some filtering options: gender, age group, diagnose period, tobacco use, region, rurality, and socioeconomic group.

### Ethical issues

We obtained the approval from Consorci Sanitari Integral Ethics Committee (16/457) and IDIAP Jordi Gol Ethics Committee (P17/207) to conduct the study. IDIAP Jordi Gol provided the data and Consorci Sanitari Integral is the institution to which the first author is affiliated and that received funding to perform the research.

Because of the observational, retrospective and population-based design, it was not possible to obtain the informed consent from patients to use their clinical information. The data used for the analysis were completely anonymized with no variables that might allow identification of individual patients. We followed the Spanish law on the protection of personal data (LOPD 15/1999 of December 14).

## Results

**Figure 1:**
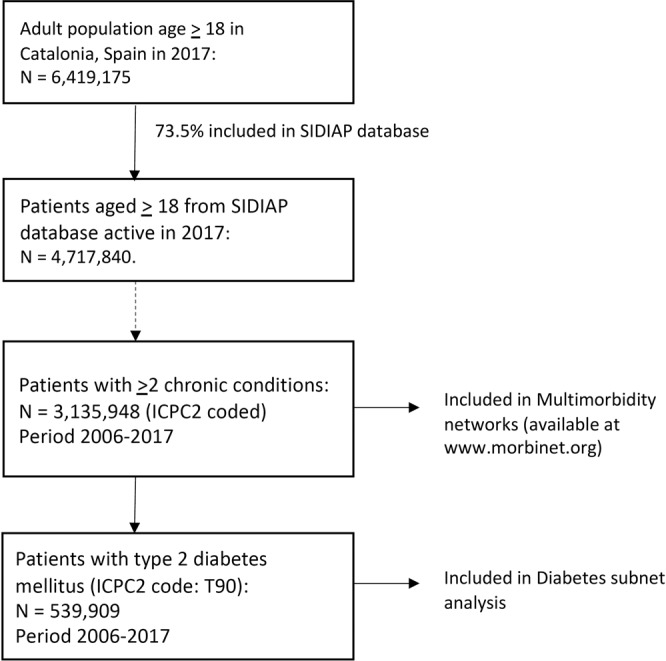
Study population. We included the information of 3,135,948 adult patients with multimorbidity (539,909 of them with T2DM) from SIDIAP database, which contains clinical data from 73.5% of population in Catalonia, Spain.

We included the information of 3,135,948 patients with multimorbidity; 539,909 of them with T2DM (Fig. 1). We used the data from 2006 to 2017 to study disease associations and construct the networks, but we used 2017 information to calculate prevalences. In 2017 the prevalence of multimorbidity in patients aged 18 and over was 52.8% for chronic conditions coded with the ICPC-2 system. In Supplementary Table S2 we present it for different age and sex groups. It was higher in women for all age groups and it increased with age: 94.2% of patients aged 70 or more have multimorbidity.

The general multimorbidity undirected network using ICD-10 code system with 3 digits and minimum OR 1.2 had 323 nodes and 7159 edges, using ICPC-2 system it had 148 nodes and 2766 edges (Supplementary Table S3) and with the simplified ICPC-2 version it had 111 nodes and 1607 edges. Multimorbidity networks and subnetworks for specific chronic conditions can be visualized at the open website^25^.

The total prevalence of T2DM (ICPC-2 code: T90) in 2017 was 7.9% (8.8% in men and 6.9% in women). In the study period T2DM was present in 20.7% of men and 14.4% of women with multimorbidity (Supplementary Table S2). This percentage varied according to the age group considered and was highest in older patients and lower socioeconomic groups, though it was similar in rural and urban areas. In men with T2DM, ex-smoker was the more frequent category in tobacco use while most diabetic women were non-smokers. Tobacco use was still present in 24.15% of men and 7.4% of women with T2DM (Supplementary Table S4).

**Figure 2:**
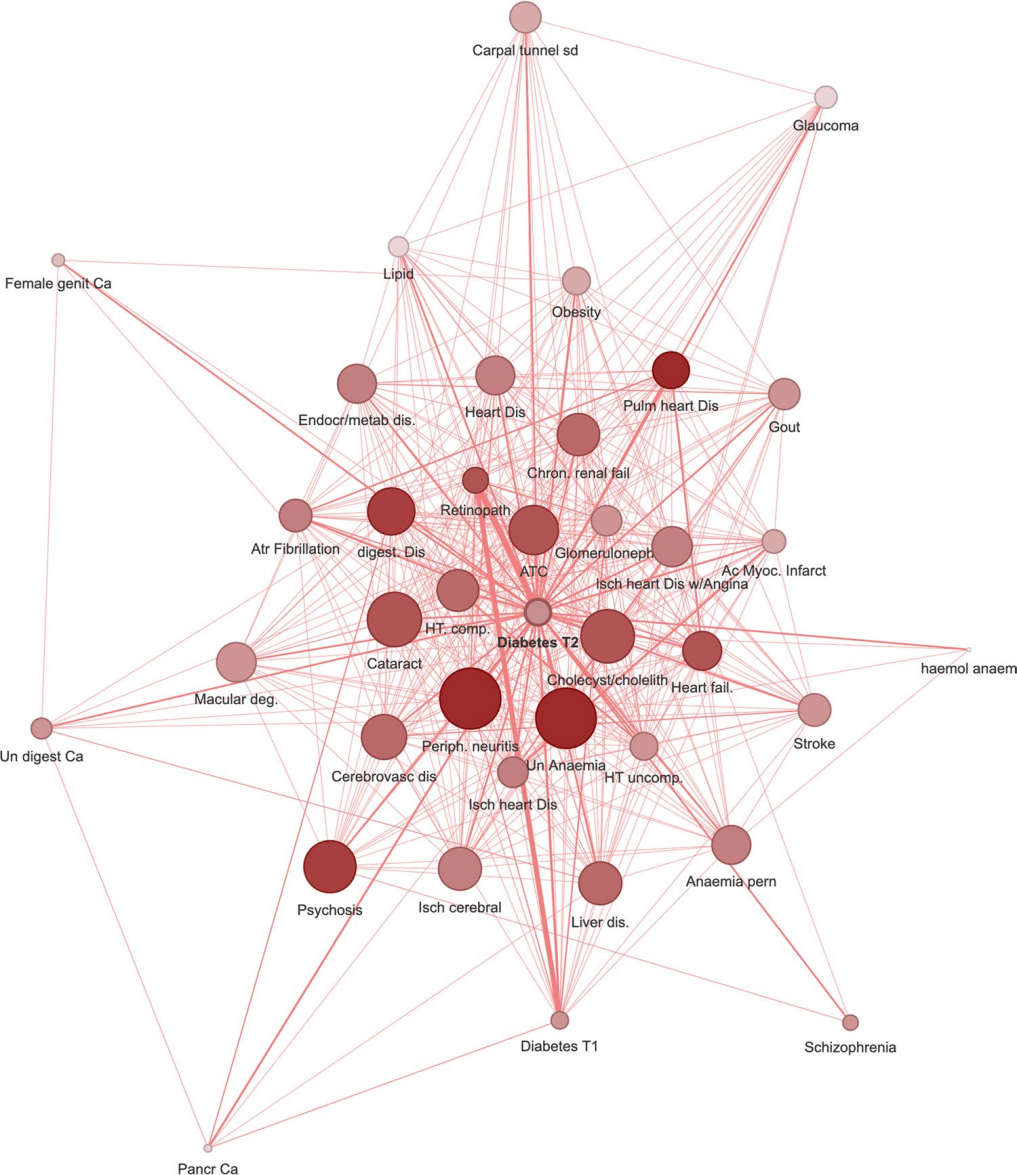
Type 2 diabetes mellitus comorbidity undirected network (OR > 1.2). It represents chronic conditions associated with an OR > 1.2. The node size shown is proportional to the degree and the colour to the pageRank index (both in the global multimorbidity network). The edges thickness is related to the OR value for the conditions connected. Thus, a big node with a dark colour has many connections and is very central in the general multimorbidity network. Abbreviations: Dis: disease; Ca: cancer; Un: unspecified. T1D: type 1 diabetes mellitus, Isch: ischemic; Ac Myoc Infarct: acute myocardial infarction; Chron: chronic; Pulm: pulmonary; HT comp: hypertension complicated; Trans: transient; ATC: atherosclerosis; fail: failure; Atr.: atrial, deg: degeneration. Figure created with the MorbiNet application v2.2^25^, https://morbinet.org/shiny.

Figure 2 represents T2DM (T90 ICPC-2 code) comorbidity undirected network with chronic conditions associated with an OR ≥ 1.2. It had 38 nodes and 448 edges (see network parameters in Supplementary Table S3). The degrees of the nodes in T2DM subnets are listed in Table 1. The conditions with more connections in non-directed networks were hypertension complicated and atherosclerosis/peripheral vascular disease (both with degree 32), and cholecystitis/cholelithiasis, retinopathy and peripheral neuritis (all with degree 31). The OR values for all pairs of connected nodes are shown in Supplementary Table S5.

**Table 1:** Degree of nodes in type 2 diabetes mellitus subnets. Abbreviations: Dis: disease, Ca: cancer, NOS: not otherwise specified.

		Non-Directed OR ≥ 1.2, Fig. 2	Directed OR ≥ 1.5, Fig. 3a		Directed OR ≥ 2, Fig. 3b	
		degree	in-degree	out-degree	in-degree	out-degree
T90	Type 2 diabetes mellitus	37	12	20	4	9
K87	Hypertension complicated	32	12	8		
K92	Atherosclerosis/peripheral vascular dis	32	14	13	7	4
D98	Cholecystitis/cholelithiasis	31				
F83	Retinopathy	31	11	16	8	6
N94	Peripheral neuritis/neuropathy	31	11	10	5	3
B82	Anaemia other, unspecified	30	12	10		
U88	Glomerulonephritis/nephrosis	30	16	13	6	4
F92	Cataract	29				
K74	Ischaemic heart dis. with angina	29	8	13		
K76	Ischaemic heart dis. without angina	29	13	9	4	3
U99.01	Chronic renal failure	29	15	10		
D99	Dis. digestive system, other	28	14	7		
K86	Hypertension uncomplicated	28	7	14	3	3
K77	Heart failure	27	15	10		
K84	Heart dis. other	27	7	6		
K91	Cerebrovascular dis.	27				
K89	Transient cerebral ischaemia	26				
K82	Pulmonary heart dis.	25	17	6		
B81	Anaemia vitamin B12/folate deficiency	24				
D97	Liver dis. NOS	24	5	5		
K75	Acute myocardial infarction	24	11	10	4	2
K78	Atrial Fibrillation/flutter	24				
T99	Endocrine/metabolic/nutrition dis. other	24				
T83/82	Obesity/overweight	23	3	10	1	3
T92	Gout	23				
K90	Stroke	22	7	8		
T89	Type 1 diabetes mellitus	22	2	15	2	8
F84	Macular degeneration	21				
P71	Psychosis organic, other	21				
T93	Lipid disorder	21	4	6		
N93	Carpal tunnel syndrome	15				
F93	Glaucoma	14	3	0		
D77	Digestive ca. other/NOS	10				
X77	Female genital ca., other	10				
D76	Pancreas ca.	7	2	2	2	1
B78	Hereditary haemolytic anaemia	5				
P72	Schizophrenia	4				

The degree, PageRank and position the nodes of T2DM subnet have in the general multimorbidity network (calculated with all diseases), are shown in Supplementary Table S6. Some nodes of the T2DM subnet, such as peripheral neuritis/neuropathy, anaemia unspecified and diseases of digestive system, have the highest number of connections and are very central in the global multimorbidity network.

**Figure 3:**
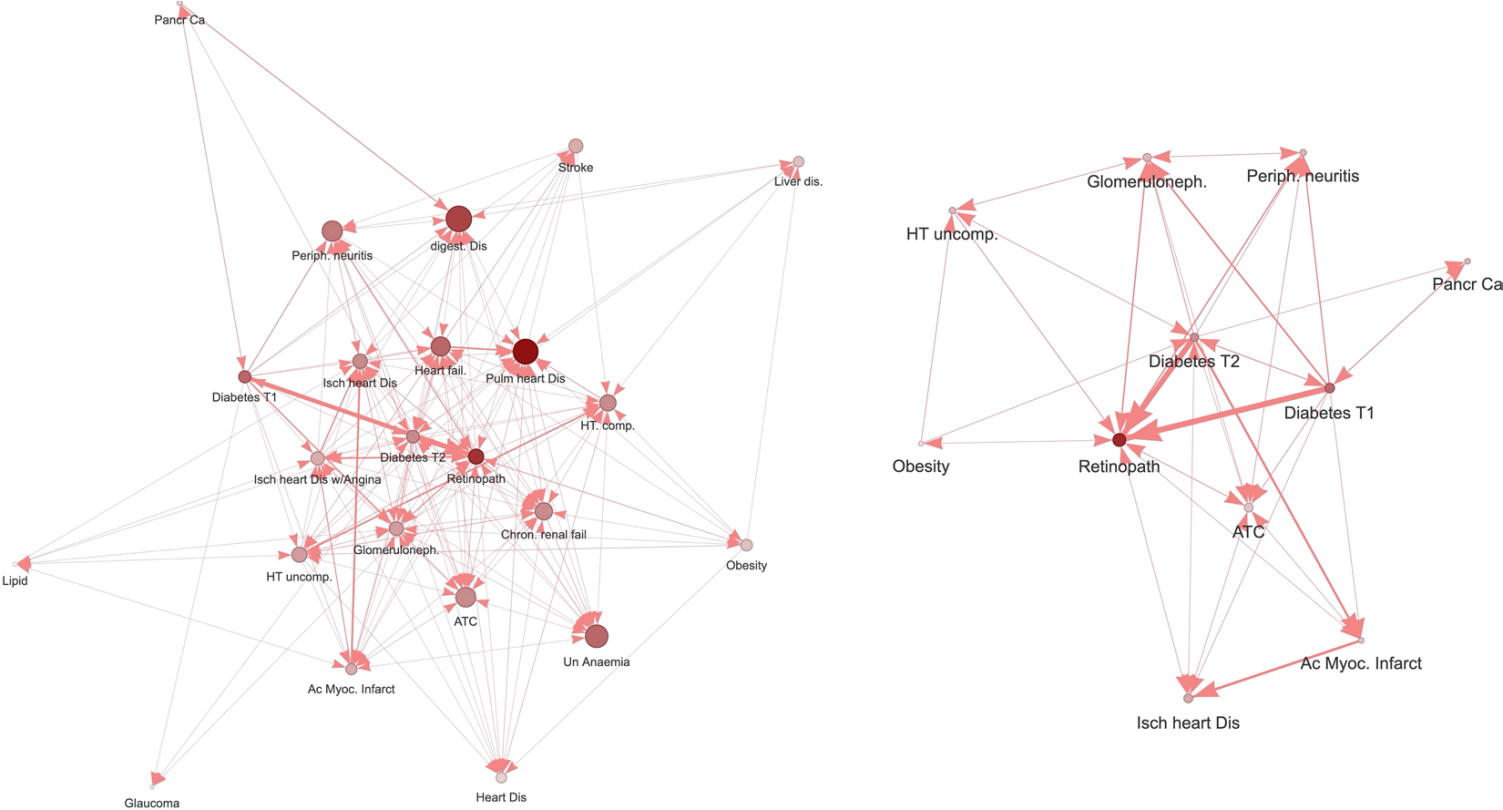
Type 2 diabetes mellitus comorbidity directed network for OR ≥ 1.5 (3a, left) and OR ≥ 2 (3b, right). The directed T2DM networks considers the temporal associations, filtered to show only chronic conditions with an OR ≥ 1.5 or ≥ 2 (stronger associations). In the cases where the temporal direction is not clear, the edges are represented with a double arrow (in both directions). The strongest associations from T2DM are to retinopathy, glomerulonephritis/nephrosis, peripheral neuritis/neuropathy and pancreas cancer. T2DM receives the strongest connections from obesity/overweight. Figures created with the MorbiNet application v2.2^25^, https://morbinet.org/shiny.

Figure 3a represents the directed T2DM network, considering the temporal associations, filtered to show only chronic conditions with an OR ≥ 1.5. It had 23 nodes and 221 edges (Supplementary Table S3). In the cases where the temporal direction is not clear, the edges are represented with a double arrow (in both directions). The edges with undefined direction are counted twice and for this reason, the number of edges is higher in directed networks as compared to undirected for the same minimum OR value. Table 2 includes the OR values for the nodes with direct links with T2DM in directed networks. The strongest associations from T2DM were to: retinopathy (OR: 23.83), glomerulonephritis/nephrosis (OR: 3.44), peripheral neuritis/neuropathy (OR: 2.70) and pancreas cancer (OR: 2.42). T2DM received the strongest connections from obesity/overweight (OR: 2.58) and ischemic heart disease with angina (1.89). The OR values for all pairs of connected nodes in each direction are shown in Supplementary Table S7. The nodes in Fig. 3b are the conditions with an OR ≥ 2 in a directed network. This network restricted to stronger associations had 11 nodes and 46 edges (Supplementary Table S3). Table 1 also included the degree of nodes in the directed T2DM subnets (in-degree or number of connections received and out-degree or connections from the considered node), both with OR ≥ 1.5 and OR > 2.

**Table 2:** Odds ratio values for T90 direct links in type 2 diabetes mellitus directed network.

From	To	Disease connected with type 2 diabetes	OR
T90	F83	Retinopathy	23.8
T90	U88	Glomerulonephritis/nephrosis	3.4
T90	N94	Peripheral neuritis/neuropathy	2.7
T90	D76	Pancreas Ca.	2.4
T90	K75	Acute myocardial infarction	2.1
T90	K92	Atherosclerosis/peripheral vascular dis	2.0
T90	K87	Hypertension complicated	1.7
T90	K82	Pulmonary heart dis.	1.6
T90	K84	Heart dis. other	1.6
T90	F93	Glaucoma	1.5
T90	K91	Cerebrovascular dis.	1.4
T90	T92	Gout	1.3
T90	F84	Macular degeneration	1.2
T83/82	T90	Obesity/overweight	2.6
K74	T90	Ischaemic heart dis. with angina	1.9
X77	T90	Female genital ca, other	1.4
K78	T90	Atrial Fibrillation/flutter	1.4
P72	T90	Schizophrenia	1.3
K89	T90	Transient cerebral ischaemia	1.3
P71	T90	Psychosis organic, other	1.2
**Undefined direction**			
T90	T89	Diabetes insulin dependent	3.9
T90	K86	Hypertension uncomplicated	2.8
T90	K76	Ischaemic heart dis. without angina	2.2
T90	D97	Liver dis. NOS	1.9
T90	K77	Heart failure	1.9
T90	U99.01	Chronic renal failure	1.8
T90	B82	Anaemia other, unspecified	1.7
T90	T93	Lipid disorder	1.7
T90	K90	Stroke	1.6
T90	D99	Dis. digestive system, other	1.6
T90	B81	Anaemia vitamin B12/folate deficiency	1.4
T90	F92	Cataract	1.4
T90	D98	Cholecystitis/cholelithiasis	1.3
T90	T99	Endocrine/metabolic/nutrition dis. other	1.3
T90	N93	Carpal tunnel syndrome	1.2
T90	D77	Digestive ca. other/NOS	1.2
T90	B78	Hereditary haemolytic anaemia	1.2

T90: Type 2 diabetes mellitus. Abbreviations: Dis: disease, Ca: cancer, NOS: not otherwise specified.

**Figure 4:**
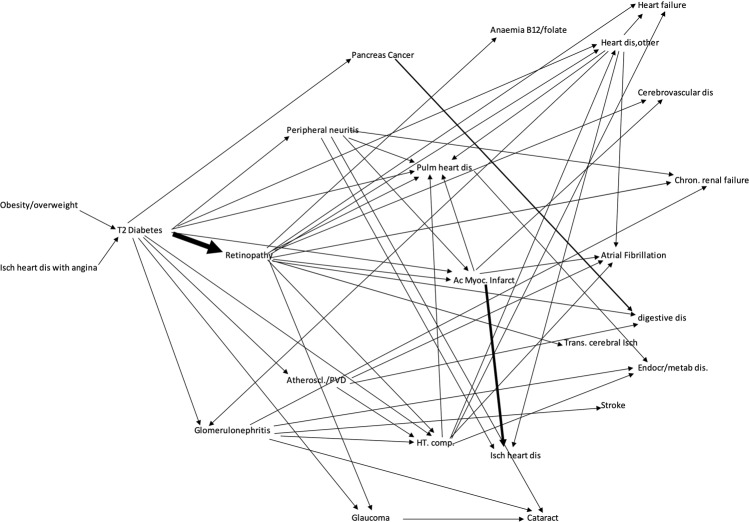
Temporal comorbid associations. We present the trajectories for T2DM, including one-step previous to diabetes and two steps from it with strong associations (OR ≥ 1.5). The thickness of the arrows is proportional to the OR value. The important role of retinopathy can be visualized. Abbreviations: dis: disease; Isch: ischemic; T2: type 2; Ac Myoc Infarct: acute myocardial infarction; chron: chronic; Pulm: pulmonary; HT comp: hypertension complicated; Trans: transient; Atheroscl: atherosclerosis; PVD: peripheral vascular disease. Figure elaborated by authors with data exported from the MorbiNet application v2.2^25^, https://morbinet.org/shiny.

In Fig. 4 we present the trajectories for T2DM, including one-step previous to diabetes and two steps from it with OR ≥ 1.5. The thickness of the arrows is proportional to the OR value. Again, the important role of retinopathy can be visualized. Some chronic conditions could be reached through different paths. Pulmonary heart disease could be directly reached (Supplementary Table S8) from 10 different previous conditions considering the trajectories up to three steps from T2DM and OR ≥ 1.5, including retinopathy, ischemic heart disease, other heart diseases, complicated hypertension, peripheral neuritis, heart failure and atrial fibrillation. Other conditions with a high number of different previous connected diseases were: atrial fibrillation/flutter and other diseases of digestive system (both with 7 different previous diseases connecting them), ischemic heart disease without angina (6 different diseases) and hypertension complicated, heart failure, chronic renal failure, and peripheral neuritis (all with 5 previous different immediate disease connections).

As expected, the number of nodes and significant interactions increased in T2DM networks when we reduced the threshold OR value (Supplementary Table S9). For OR 2.0 there were only 11 nodes and 32 links, for OR 1.5 the network had 23 nodes and 156 links and for OR 1.2 it had 38 nodes and 439 links. For other OR values see Supplementary Table S9. All networks can be visualized in the website.

If we only included the patients with more complete data in T2DM networks the number of nodes and interactions were slightly reduced, which can be explained because the number of patients was lower and so was the statistical power. For OR 1.5, when all data were included the network had 23 nodes and 156 interactions and when we included the data with higher quality we had the same number of nodes and 150 links. For other OR values see Supplementary Table S9. Thus, this variable did not seem to have a great impact on the results.

The criterion to define direction of temporal associations in T2DM was arbitrarily set at < 40% or > 60% of previous/subsequent diagnosis among pairs of chronic conditions. When this criterion was changed to a more stringent threshold of < 20% or > 80%, there was an important reduction on the number of temporal associations identified. As can be seen in Supplementary Table S10, the number of directional associations were reduced 80% on average, similar for different OR values. This means most directional interactions for pairs of chronic conditions of T2DM networks were between probabilities of 20–40% or 60–80%. In Supplementary table S11 we included the list of directional associations using the < 20% or 80% criteria in T2DM network.

## Discussion

We have estimated multimorbidity associations in the general adult population and developed an interactive tool to construct and visualize them graphically, with network parameters. We used a large database, including the information of 3,135,948 adult people with multimorbidity recorded in a long period (12 years), which also allowed estimating temporal direction of the associations. This interactive application can be customized to different needs. Networks can be constructed using different classification systems and can be used both for primary or specialized care. The burden of disease should be considered globally. However, usually in the clinical practice, there is a first chronic disease that occurs, or physicians may be interested in one specific disease because it is more serious or require a more complex treatment. For this reason, it might be useful to extract the comorbidity network for that condition and visualize the diseases associated. This approach might help to analyse the multimorbidity network and to adapt it to clinical practice situations. It is possible to extract subnets for 324 chronic conditions with the ICD-10 system, and 148 chronic diseases using ICPC-2 classification. Apart from the multimorbidity network adjusted by sex and age, stratified networks for gender and different age groups can also be plotted. With this application, both health professionals and the general public can visualize and explore the relationships between diseases according to their specific interests.

We have analysed in detail the T2DM comorbidity network. Most of the conditions T2DM is connected to (Table 2, Fig. 3) are well-known complications of diabetes: retinopathy, renal disease, neuropathy, and cardiovascular disease. T2DM is an important risk factor for cardiovascular events, which is a leading cause of mortality in diabetic patients^26^, Essential hypertension was reported to play an important role in the association between T2DM and comorbid diseases, such as stroke and dyslipidaemia^27^ and it is an important risk factor for dying from diabetes^28^.

Retinopathy reflects the damage to microvasculature and is a marker for cerebral vascular disease^29^ and system diabetic vasculopathy^30^. Patients with severe stages of retinopathy (macular oedema or proliferative retinopathy) have an increased risk of developing cardiovascular disease^31^. Our results confirm patients with T2DM also have a higher risk of cataracts^32^ and open-angle glaucoma^33^.

We found T2DM to be associated with liver diseases, which includes non-alcoholic fatty liver disease. When they both coexist, the risk of complications of diabetes and more severe liver disease increases^34^. Non-alcoholic fatty liver disease is linked with insulin resistance which affects glucose and lipid metabolism, increasing lipogenesis and gluconeogenesis^35^. Diabetes has been independently associated with an increased incidence of anaemia of chronic diseases, which is normochromic and normocytic in the context of inflammatory states^36^. Anaemia is associated with a higher risk of death in patients with T2DM^37^. Atrial fibrillation-flutter also connects to T2DM (Table 2). This association has also been reported in several studies^38^.

Alonso-Moran *et al*. conducted a study in a large data set of patients with T2DM and found more risk in 21 out of 51 chronic diseases^39^. Our OR values are higher, which might be in part because they included older patients. They found an increased risk for emphysema-chronic bronchitis, depression, and psoriasis-eczema, but with OR values were below 1.2. This might be the reason why we did not find these associations. They obtained a reduced risk for malignancies (overall), while we obtained an increased risk for other gynaecological cancers (X77, which includes the uterus, ovarium, vagina, vulva, and others), other digestive cancers (D77, which includes mouth, oesophagus, small intestine, liver, biliary cancers and others) and pancreas cancer. T2DM is a known risk factor associated with pancreatic cancer^40^. A higher risk of endometrial cancer has also been observed in patients with T2DM^41^. Insulin resistance, chronic inflammation, and elevated free steroid hormones have been suggested as possible mechanisms involved. Some studies found an increased risk of gastric cancer in diabetes^42^, while others reported little or no change in the risk^43^.

High levels of uric acid were attributed to insulin resistance because of a reduced excretion of uric acid, and recently hyperuricemia has also been reported to have a potential role in incident diabetes^44^. According to our results, T2DM would precede gout more often than otherwise.

We did not find some of the associations reported by Klime *et al*. such as Parkinson or epilepsy, whose study included persons with diabetes who had received inpatient care^45^. Our study was based in general population and we focused on diseases with strong associations, with OR values at least above 1.2, which may hide real associations but with small magnitude.

Regarding directed networks, the analysis showed that both psychosis and schizophrenia precede T2DM (Table 2). Other studies have also found these comorbidities^39,45^. Lifestyle risk factors for diabetes were common in patients with schizophrenia (sedentarism, poor diet, obesity) and atypical neuroleptics have been associated with T2DM^46^.

Carpal tunnel syndrome was associated with T2DM. A meta-analysis suggests that diabetes is a risk factor for carpal tunnel syndrome, and the mechanisms of this increased risk are still being investigated^47^.

These networks show association, and even in the directed networks no causal link should be attributed, since diseases might be associated because they share a risk factor, pathogeny pathway, genetic links or might be caused or influenced by medication (adverse events or protective effect). Many chronic diseases might share some common underlying mechanisms which are driven by oxidative stress^48^.

A prevalent disease can coexist with T2DM, but it can be present no more frequently than in patients without diabetes. The networks we constructed show associations greater than those expected because of their prevalence. Some studies describe the most common comorbidities of T2DM, without considering the prevalence of diseases or having a control group and give complementary information to our results^49–51^.

Networks offer a more global picture because it includes not only direct connections but also indirect associations which give more accurate information about comorbidity. T2DM is not one of the chronic diseases with more connections. In the multimorbidity network, with ICPC-2 nodes and OR ≥1.2, the degree is 37 with position 80 out of 148. It is not very central either, its centrality PageRank index is in position 65/148. However, it is connected to nodes with a very high degree and very central in the global multimorbidity network and some of its connections include severe chronic conditions with a poor prognosis. PageRank gives information about the node centrality in the global network. A node with a high value is very connected directly or through other nodes. It connects different clusters or communalities. Pulmonary heart disease has the second-highest PageRank value in the multimorbidity network, although its prevalence is very low. Some conditions with higher prevalence are very central and have a lot of connections in the global multimorbidity network: neuropathy (highest number of connections and 4^th^ position in centrality), anaemia (2^nd^ in degree and 5^th^ more central), and other digestive diseases (9^th^ position both in number of connections and centrality). D99, other digestive system diseases, includes vascular intestine alterations, intestinal malabsorption, celiac disease, pancreas conditions, and others. Cholecystitis/cholelithiasis and cataract have also a high degree (6^th^ and 7^th^ position out of 148) in the general network. The causal genes of central diseases, with a major impact on multimorbidity, have the potential to influence multiple diseases^52^.

Because of the long study period (2006–2017), we could analyse temporal associations and draw trajectories. They show the relevance of retinopathy in the progress of T2DM. It is not only the condition with the strongest association following T2DM, in Fig. 4 we can see how many chronic diseases have direct links with origin in retinopathy. They include severe conditions with increased disability, reduced quality of life, increased mortality and important use of health resources such as complicated hypertension, cerebrovascular disease, ischemic heart disease, pulmonary heart disease and organ failure (chronic renal failure, heart failure). We have shown the key role of retinopathy. It suggests the importance to prevent it and monitor fundus exploration for early detection. We would like to note in Fig. 4 we just included the conditions with a strong association (OR ≥ 1.5), otherwise, the picture would be even more complex. Some diseases can be reached from different pathways (Supplementary Table S8), so the risk might be higher than expected if we just looked at immediate connections. Directed networks and disease trajectories consider time and can be useful to identify risk factors and complications of diseases but also study associations to other conditions that take place earlier or later in lifetime and can affect the health status.

Jansen *et al*. studied temporal trajectories of some chronic diseases including diabetes cluster, analysing acute and chronic conditions with hospital encounters data of a large population in 14.9 years^53^. They also identified the retinal disease as a keystone diagnosis marking the progression to worse conditions.

## Limitations

We used SIDIAP database, which contains information from 73% of the Catalan population using the public health care system. Users of private healthcare, presumably with a higher socioeconomic status and probably better health situation were not included. SIDIAP database contains information from electronic medical records. They might include diagnostic errors and some conditions might have been assigned wrong codes or not be recorded in the electronic files. This could conduct to selection bias and limit in part the extrapolation of results, although the large sample size and population-based approach probably protects against major biases.

Directed networks were constructed considering when a diagnosis was made and recorded which does not necessarily imply the moment when the disease started. A condition could have been diagnosed after another one even though it might have started before, leading to wrong temporal assumptions. An additional limitation in T2DM directional networks was the fact that most of the directional interactions were not strongly defined, since 80% of them were in the range of probabilities between 20–40% or 60–80%. That means that only 20% of the temporal associations identified had 80% or more consistency (or less than 20% temporal discordances).

Since the 2014 version, the ICD-10 descriptor for code E11 is “type 2 diabetes mellitus”. But in previous versions, at the time when a lot of the patients might have been diagnosed, the descriptor for E11 was “non-insulin dependent diabetes mellitus” and as some patients with T2DM are treated with insulin this descriptor might create confusion. For this reason, the associations between type1 and type 2 diabetes should be looked with caution.

In the networks, we included diseases associated to T2DM more than what would be expected because of their prevalence. Thus, frequent diseases can also coexist with diabetes but no more often than in patients without this condition.

## Conclusions

We presented T2DM comorbidities with a strong association and so most of them have already been reported. Our study provides a visual representation based on a large general population and a long follow up period and shows not only direct associations but indirect ones as well as parameters that define the position of T2DM comorbidities in the global multimorbidity network. Neuropathy, anaemia and digestive diseases are comorbidities very connected and central in the general multimorbidity network. We also constructed trajectories that show temporal associations and identify the diseases with an important role in the progression of T2DM, such as retinopathy. Our open interactive website might be useful to explore multimorbidity in a customized way.

## Supplementary information

Supplementary information.

## Data Availability

The data that support the results of the study are available from SIDIAP. Some restrictions apply to the availability of them. They were released after the signature of a contract and can only be used for the current study, and so they are not publicly available. However, they are available from the authors upon reasonable request and with permission of SIDIAP.
