# Supplementary material for: MorbiNet: multimorbidity networks in adult general population. Analysis of type 2 diabetes mellitus comorbidity
Source: Sci Rep. 2020 Feb 12;10:2416. doi: 10.1038/s41598-020-59336-1 (PMC7016191; doi:10.1038/s41598-020-59336-1)
Supplement: Supplementary file 1 — Supplementary information. [file 41598_2020_59336_MOESM1_ESM.docx]

**MorbiNet: multimorbidity networks in adult general population. Analysis of type 2 diabetes mellitus comorbidity**

**Alba Aguado, Ferran Moratalla-Navarro, Flora López-Simarro, Victor Moreno**

Supplementary information

Table S1. List of chronic conditions with ICPC2 simplified version.

Table S2. Prevalence of multimorbidity (ICPC-2 coded chronic conditions) and T2DM (T90 code) in 2017.

Table S3. Network parameters. Code system: ICPC2.

Table S4. Age group, tobacco use, rurality and socioeconomic index in patients with type 2 diabetes mellitus from the total population with multimorbidity (period: 2006-2017).

Table S5. Odds Ratio values for all connected nodes in T2DM undirected network (OR > 1.2).

Table S6. Degree and PageRank of T2DM nodes in the global multimorbidity network.

Table S7. Odds ratio values for all nodes connected in directed diabetes network (for OR >1.2).

Table S8. Temporal comorbid associations. Conditions receiving immediate connections from different diseases in T2DM trajectories.

Table S9. Sensitivity analysis for T2DM networks. Whole population (ICPC-2) versus patients with more complete data (ICPC-2 HQ) for different threshold OR values

Table S10. Sensitivity analysis for T2DM networks. Temporal association criteria: probabilities of <40% or >60% versus <20% or >80%of previous/subsequent diagnosis among pairs of chronic conditions.

Table S11. Directional associations in T2DM directed networks using <20% or >80% criteria.

**Table S1. List of chronic conditions with ICPC2 simplified version.**

| **ICPC 2 code** | **Description** | **Codes and grouped codes in ICPC2 simplified version** |
| --- | --- | --- |
| ***General and unspecified*** | | |
| **A04** | Weakness/tiredness general | A04 |
| **A70** | Tuberculosis | A70 |
| **A79** | Malignancy NOS | A79 |
| **A90** | Congenital anomaly OS/multiple | A90 |
| ***Blood, Blood Forming Organs and Immune Mechanism*** | | |
| **B75** | Benign/unspecified neoplasm blood | B75 |
| **B83** | Purpura/coagulation defect | B83 |
| **B90** | HIV-infection/aids | B90 |
| **B72** | Hodgkin's disease/lymphoma |  |
| **B73** | Leukaemia |  |
| **B74** | Malignant neoplasm blood other |  |
|  | Blood cancer / lymphoma | B72, B73, B74 |
| **B78** | Hereditary haemolytic anaemia | B78 |
| **B81** | Anaemia, Vitamin B12/folate def. |  |
| **B82** | Anaemia other/unspecified |  |
|  | Anaemia | B81, B82 |
| ***Digestive*** | | |
| **D72** | Viral hepatitis | D72 |
| **D81** | Congen. anomaly digestive system | D81 |
| **D84** | Oesophagus disease | D84 |
| **D92** | Diverticular disease | D92 |
| **D93** | Irritable bowel syndrome | D93 |
| **D94** | Chronic enteritis/ulcerative colitis | D94 |
| **D97** | Liver disease NOS | D97 |
| **D98** | Cholecystitis/cholelithiasis | D98 |
| **D99** | Disease digestive system, other | D99 |
| **D74** | Malignant neoplasm stomach |  |
| **D75** | Malignant neoplasm colon/rectum | D75 |
| **D76** | Malignant neoplasm pancreas |  |
| **D77** | Malig. neoplasm digest other/NOS |  |
|  | Digestive cancer, other | D74, D76, D77 |
| **D85** | Duodenal ulcer |  |
| **D86** | Peptic ulcer other |  |
|  | Peptic ulcer | D85, D86 |

| ***Eye*** | | |
| --- | --- | --- |
| **F74** | Neoplasm of eye/adnexa | F74 |
| **F92** | Cataract | F92 |
| **F93** | Glaucoma | F93 |
| **F94** | Blindness | F94 |
| **F82** | Detached retina |  |
| **F83** | Retinopathy |  |
| **F84** | Macular degeneration |  |
|  | Retinopathy | F82, F83, F84 |
| ***Ear*** | | |
| **H75** | Neoplasm of ear | H75 |
| **H82** | Vertiginous syndrome | H82 |
| **H84** | Presbyacusis | H84 |
| **H86** | Deafness | H86 |
| ***Cardiovascular*** | | |
| **K72** | Neoplasm cardiovascular | K72 |
| **K77** | Heart failure | K77 |
| **K88** | Postural hypotension | K88 |
| **K92** | Atherosclerosis/Peripheral vascular disease | K92 |
| **K93** | Pulmonary embolism | K93 |
| **K73** | Congenital anomaly cardiovascular | K73 |
| **K71** | Rheumatic fever/heart disease |  |
| **K81** | Heart/arterial murmur NOS | K81 |
| **K82** | Pulmonary heart disease | K82 |
| **K83** | Heart valve disease NOS |  |
| **K84** | Heart disease, other | K84 |
|  | Rheumatic/heart valve disease | K71, K83 |
| **K74** | Ischaemic heart disease w. angina |  |
| **K75** | Acute myocardial infarction |  |
| **K76** | Ischaemic heart disease without angina |  |
|  | Ischemic heart disease | K74, K75, K76 |
| **K78** | Atrial fibrillation/flutter |  |
| **K79** | Paroxysmal tachycardia |  |
| **K80** | Cardiac arrhythmia NOS |  |
|  | Cardiac arrhythmia | K78, K79, K80 |
| **K86** | Hypertension uncomplicated |  |
| **K87** | Hypertension complicated |  |
|  | Hypertension | K86, K87 |
| **K89** | Transient cerebral ischaemia |  |
| **K90** | Stroke/cerebrovascular accident |  |
| **K91** | Cerebrovascular disease |  |
|  | Cerebrovascular disease | K89, K90, K91 |
| **K94** | Phlebitis/thrombophlebitis |  |
| **K95** | Varicose veins of leg |  |
|  | Phebitis/varicose veins | K94, K95 |
| ***Musculoskeletal*** | | |
| **L71** | Malignant neoplasm musculoskeletal | L71 |
| **L85** | Acquired deformity of spine | L85 |
| **L88** | Rheumatoid/seropositive arthritis | L88 |
| **L92** | Shoulder syndrome | L92 |
| **L93** | Tennis elbow | L93 |
| **L95** | Osteoporosis | L95 |
| **L82** | Congenital anomaly musculoskeletal | L82 |
| **L99** | Musculoskeletal disease other | L99 |
| **L83** | Neck syndrome |  |
| **L84** | Back syndrome without radiating pain |  |
| **L86** | Back syndrome with radiating pain |  |
|  | Neck/back syndrome | L83, L84, L86 |
| **L89** | Osteoarthrosis of hip |  |
| **L90** | Osteoarthrosis of knee |  |
| **L91** | Osteoarthrosis other |  |
|  | Osteoarthrosis | L89, L90, L91 |
| ***Neurological*** |  |  |
| **N73** | Neurological infection, other | N73 |
| **N74** | Malignant neoplasm nervous system | N74 |
| **N86** | Multiple sclerosis | N86 |
| **N87** | Parkinsonism | N87 |
| **N88** | Epilepsy | N88 |
| **N92** | Trigeminal neuralgia | N92 |
| **N75** | Benign neoplasm nervous system |  |
| **N76** | Neoplasm nervous system unspec. |  |
|  | Benign/unspecified nervous system neoplasm | N75, N76 |
| **N85** | Congenital anomaly neurological | N85 |
| **N89** | Migraine |  |
| **N90** | Cluster headache |  |
|  | Migraine/headache | N89, N90 |
| **N93** | Carpal tunnel syndrome | N93 |
| **N94** | Peripheral neuritis/neuropathy | N94 |
| N99 | Neurological disease, other | N99 |
| ***Psychological*** | | |
| **P15** | Chronic alcohol abuse | P15 |
| **P17** | Tobacco abuse | P17 |
| **P19** | Drug abuse | P19 |
| **P70** | Dementia | P70 |
| **P78** | Neuraesthenia/surmenage | P78 |
| **P79** | Phobia/compulsive disorder | P79 |
| **P81** | Hyperkinetic disorder | P81 |
| **P85** | Mental retardation | P85 |
| **P86** | Anorexia nervosa/bulimia | P86 |
| **P71** | Organic psychosis other |  |
| **P73** | Affective psychosis |  |
| **P98** | Psychosis NOS/other |  |
|  | Psychosis | P71, P73, P98 |
| **P72** | Schizophrenia | P72 |
| **P80** | Personality disorder | P80 |
| **P74** | Anxiety disorder/anxiety state |  |
| **P75** | Somatization disorder |  |
| **P82** | Post-traumatic stress disorder |  |
|  | Anxiety/somatization/post-traumatic stress | P74, P75, P82 |
| **P76** | Depressive disorder | P76 |
| ***Respiratory*** | | |
| **R90** | Hypertrophy tonsils/adenoids | R90 |
| **R96** | Asthma | R96 |
| **R95** | Chronic obstructive pulmonary disease | R95 |
| **R84** | Malignant neoplasm bronchus/lung | R84 |
| **R85** | Malignant neoplasm respiratory, other | R85 |
| **R99** | Respiratory disease, other | R99 |
| ***Skin*** | | |
| **S77** | Malignant neoplasm of skin | S77 |
| **S91** | Psoriasis | S91 |
| **S96** | Acne | S96 |
| **S86** | Dermatitis seborrhoeic |  |
| **S87** | Dermatitis/atopic eczema |  |
|  | Dermatitis/atopic eczema | S86, S87 |
| **S99** | Skin disease, other | S99 |
| ***Endocrine/Metabolic and Nutritional*** | | |
| **T71** | Malignant neoplasm thyroid | T71 |
| **T73** | Neoplasm endocrine oth/unspecified | T73 |
| **T80** | Congenital anom endocrine/metab. | T80 |
| **T81** | Goitre | T81 |
| **T85** | Hyperthyroidism/thyrotoxicosis | T85 |
| **T86** | Hypothyroidism/myxoedema | T86 |
| **T92** | Gout | T92 |
| **T93** | Lipid disorder | T93 |
| **T80** | Congenital anom endocrine/metab. | T80 |
| **T99** | Endocrine/metabolic/nutritional disease other | T99 |
| **T82** | Obesity |  |
| **T83** | Overweight |  |
|  | Overweight/obesity | T82, T83 |
| **T89** | Diabetes insulin dependent |  |
| **T90** | Diabetes non-insulin dependent |  |
|  | Diabetes | T89, T90 |
| ***Urological*** |  |  |
| **U88** | Glomerulonephritis/nephrosis | U88 |
| **U99** | Urinary disease, other | U99 |
| **U99.01** | Chronic renal failure | U99.01 |
| **U75** | Malignant neoplasm of kidney |  |
| **U76** | Malignant neoplasm of bladder |  |
| **U77** | Malignant neoplasm urinary other |  |
|  | Urinary cancer | U75, U76, U77 |
| ***Pregnancy, Childbearing, Family Planning*** | | |
| **W15** | Infertility/subfertility | W15 |
| **W72** | Malignant neoplasm related to pregnancy | W72 |
| ***Female Genital*** | | |
| **X74** | Pelvic inflammatory disease | X74 |
| **X75** | Malignant neoplasm cervix |  |
| **X76** | Malignant neoplasm breast female | X76 |
| **X77** | Malignant neoplasm genital other |  |
|  | Genital female cancer | X75, X77 |
| **X99** | Genital disease, other | X99 |
| ***Male Genital*** | | |
| **Y85** | Benign prostatic hypertrophy | Y85 |
| **Y77** | Malignant neoplasm prostate |  |
| **Y78** | Malign neoplasm male genital other |  |
|  | Male genital cancer | Y77, Y78 |

**Table S2. Prevalence of multimorbidity (ICPC-2 coded chronic conditions) and T2DM (T90 code) in 2017.**

|  | Women | | | | Men | | | | Total | | | |
| --- | --- | --- | --- | --- | --- | --- | --- | --- | --- | --- | --- | --- |
|  | Multimorbidity | | T2DM | | Multimorbidity | | T2DM | | Multimorbidity | | T2DM | |
| Age-group | N | % | N | % | N | % | N | % | N | % | N | % |
| 18-49 | 412803 | 33.1% | 7812 | 0.6% | 317811 | 24.4% | 13447 | 1.0% | 730614 | 28.7% | 21259 | 0.8% |
| 50-69 | 542122 | 76.9% | 56689 | 8.0% | 476195 | 70.5% | 94063 | 13.9% | 1018317 | 73.8% | 150752 | 10.9% |
| 70+ | 437387 | 94.4% | 103287 | 22.3% | 305261 | 93.8% | 95504 | 29.4% | 742648 | 94.2% | 198791 | 25.2% |
| Total | 1392312 | 57.6% | 167788 | 6.9% | 1099267 | 47.8% | 203014 | 8.8% | 2491579 | 52.8% | 370802 | 7.9% |

**Table S3. Network parameters. Code system: ICPC2.**

|  | Nodes | Edges | Diameter | Density | Clustering coefficient | Centralization |
| --- | --- | --- | --- | --- | --- | --- |
| OR > 1.2 |  |  |  |  |  |  |
| Multimorbidity undirected network | 148 | 2766 | 4 | 0.25 | 0.58 | 0.34 |
| Multimorbidity directed network | 148 | 3979 | 5 | 0.18 | 0.58 | 0.28 |
| Type 2 Diabetes undirected network | 38 | 448 | 2 | 0.64 | 0.8 | 0.36 |
| Type 2 Diabetes directed network | 38 | 632 | 4 | 0.45 | 0.81 | 0.29 |
| OR > 1.5 |  |  |  |  |  |  |
| Multimorbidity undirected network | 147 | 1277 | 4 | 0.12 | 0.44 | 0.2 |
| Multimorbidity directed network | 147 | 1687 | 6 | 0.08 | 0.45 | 0.14 |
| Type 2 Diabetes undirected network | 23 | 156 | 2 | 0.62 | 0.82 | 0.38 |
| Type 2 Diabetes directed network | 23 | 221 | 3 | 0.44 | 0.82 | 0.3 |
| OR > 2 |  |  |  |  |  |  |
| Multimorbidity undirected network | 143 | 525 | 9 | 0.05 | 0.45 | 0.1 |
| Multimorbidity directed network | 143 | 715 | 11 | 0.04 | 0.45 | 0.08 |
| Type 2 Diabetes undirected network | 11 | 32 | 2 | 0.58 | 0.83 | 0.42 |
| Type 2 Diabetes directed network | 11 | 46 | 4 | 0.42 | 0.83 | 0.31 |

In the case of directed networks, the edges with undefined direction are counted twice (for each of the directions, in-degree and out-degree). For this reason, density is lower in directed networks because it has more edges per node.

**Table S4. Age group, tobacco use, rurality and socioeconomic index in patients with type 2 diabetes mellitus from the total population with multimorbidity (period: 2006-2017).**

|  | Men | | | Women | | |
| --- | --- | --- | --- | --- | --- | --- |
|  | T90 | Multimorbidity | % | T90 | Multimorbidity | % |
| Age group |  |  |  |  |  |  |
| 18-49 | 41,270 | 550,071 | 7.5% | 23,678 | 671,588 | 3.5% |
| 50-69 | 147,661 | 543,744 | 27.2% | 99,111 | 597,801 | 16.6% |
| 70+ | 101,921 | 314,166 | 32.4% | 126,268 | 458,578 | 27.5% |
| Tobacco use |  |  |  |  |  |  |
| non-smoker | 96,338 | 484,756 | 19.9% | 197,092 | 1,108,391 | 17.8% |
| smoker | 70,232 | 445,612 | 15.8% | 18,439 | 315,059 | 5.9% |
| ex-smoker | 116,250 | 399,090 | 29.1% | 24,119 | 197,700 | 12.2% |
| Rurality |  |  |  |  |  |  |
| rural | 56,329 | 271,438 | 20.8% | 45,220 | 307,301 | 14.7% |
| urban | 234,137 | 1,135,043 | 20.6% | 203,363 | 1,418,371 | 14.3% |
| Socioeconomic level |  |  |  |  |  |  |
| 1 | 36,733 | 193,692 | 19.0% | 29,815 | 258,294 | 11.5% |
| 2 | 39,429 | 199,522 | 19.8% | 33,050 | 254,512 | 13.0% |
| 3 | 41,927 | 202,599 | 20.7% | 35,542 | 253,184 | 14.0% |
| 4 | 43,138 | 204,225 | 21.1% | 37,754 | 251,875 | 15.0% |
| 5 | 42,682 | 197,107 | 21.7% | 38,875 | 230,978 | 16.8% |
| Total | 290,852 | 1,407,981 | 20.7% | 249,057 | 1,727,967 | 14.4% |

T90 ICPC2 code: type 2 diabetes mellitus. Socioeconomic level (1: higher, 5: lower)

**Table S5. Odds Ratio values for all connected nodes in T2DM undirected network (OR > 1.2).**

| ICPC2 code | Description | ICPC2 code | Description | OR |
| --- | --- | --- | --- | --- |
| B82 | Anaemia other, unspecified | B78 | Hereditary haemolytic anaemia | 2.097 |
| K82 | Pulmonary heart dis. | B78 | Hereditary haemolytic anaemia | 1.609 |
| B81 | Anaemia vitamin B12/folate deficiency | B78 | Hereditary haemolytic anaemia | 1.489 |
| D98 | Cholecystitis/cholelithiasis | B78 | Hereditary haemolytic anaemia | 1.245 |
| K82 | Pulmonary heart dis. | B81 | Anaemia vitamin B12/folate deficiency | 1.424 |
| K82 | Pulmonary heart dis. | B82 | Anaemia other, unspecified | 2.316 |
| B81 | Anaemia vitamin B12/folate deficiency | B82 | Anaemia other, unspecified | 2.101 |
| T89 | Type 1 Diabetes mellitus | B82 | Anaemia other, unspecified | 1.397 |
| F84 | Macular degeneration | B82 | Anaemia other, unspecified | 1.223 |
| D76 | Pancreas Ca. | D77 | Digestive ca. other/NOS | 2.941 |
| B82 | Anaemia other, unspecified | D77 | Digestive ca. other/NOS | 1.481 |
| B81 | Anaemia vitamin B12/folate deficiency | D77 | Digestive ca. other/NOS | 1.243 |
| D77 | Digestive ca. other/NOS | D97 | Liver dis. NOS | 3.989 |
| D98 | Cholecystitis/cholelithiasis | D97 | Liver dis. NOS | 2.945 |
| K82 | Pulmonary heart dis. | D97 | Liver dis. NOS | 1.801 |
| T99 | Endocrine/metabolic/nutrition dis. other | D97 | Liver dis. NOS | 1.577 |
| K87 | Hypertension complicated | D97 | Liver dis. NOS | 1.545 |
| B82 | Anaemia other, unspecified | D97 | Liver dis. NOS | 1.441 |
| B81 | Anaemia vitamin B12/folate deficiency | D97 | Liver dis. NOS | 1.433 |
| D76 | Pancreas Ca. | D97 | Liver dis. NOS | 1.375 |
| F83 | Retinopathy | D97 | Liver dis. NOS | 1.307 |
| K77 | Heart failure | D97 | Liver dis. NOS | 1.283 |
| X77 | Female genital ca, other | D97 | Liver dis. NOS | 1.27 |
| F84 | Macular degeneration | D97 | Liver dis. NOS | 1.267 |
| K92 | Atherosclerosis/peripheral vascular dis | D97 | Liver dis. NOS | 1.237 |
| P71 | Psychosis organic, other | D97 | Liver dis. NOS | 1.2 |
| D76 | Pancreas Ca. | D98 | Cholecystitis/cholelithiasis | 1.821 |
| D77 | Digestive ca. other/NOS | D98 | Cholecystitis/cholelithiasis | 1.744 |
| K82 | Pulmonary heart dis. | D98 | Cholecystitis/cholelithiasis | 1.568 |
| B81 | Anaemia vitamin B12/folate deficiency | D98 | Cholecystitis/cholelithiasis | 1.478 |
| B82 | Anaemia other, unspecified | D98 | Cholecystitis/cholelithiasis | 1.32 |
| F84 | Macular degeneration | D98 | Cholecystitis/cholelithiasis | 1.303 |
| T99 | Endocrine/metabolic/nutrition dis. other | D98 | Cholecystitis/cholelithiasis | 1.296 |
| X77 | Female genital ca, other | D98 | Cholecystitis/cholelithiasis | 1.285 |
| D76 | Pancreas Ca. | D99 | Dis. digestive system, other | 5.72 |
| T89 | Type 1 Diabetes mellitus | D99 | Dis. digestive system, other | 2.939 |
| D98 | Cholecystitis/cholelithiasis | D99 | Dis. digestive system, other | 2.73 |
| D97 | Liver dis. NOS | D99 | Dis. digestive system, other | 2.209 |
| K92 | Atherosclerosis/peripheral vascular dis | D99 | Dis. digestive system, other | 2.1 |
| B82 | Anaemia other, unspecified | D99 | Dis. digestive system, other | 2.063 |
| B81 | Anaemia vitamin B12/folate deficiency | D99 | Dis. digestive system, other | 1.929 |
| D77 | Digestive ca. other/NOS | D99 | Dis. digestive system, other | 1.879 |
| K82 | Pulmonary heart dis. | D99 | Dis. digestive system, other | 1.815 |
| F83 | Retinopathy | D99 | Dis. digestive system, other | 1.7 |
| K91 | Cerebrovascular dis. | D99 | Dis. digestive system, other | 1.667 |
| K74 | Ischaemic heart dis. with angina | D99 | Dis. digestive system, other | 1.631 |
| U99.01 | Chronic renal failure | D99 | Dis. digestive system, other | 1.62 |
| K77 | Heart failure | D99 | Dis. digestive system, other | 1.615 |
| U88 | Glomerulonephritis/nephrosis | D99 | Dis. digestive system, other | 1.61 |
| T90 | Type 2 Diabetes mellitus | D99 | Dis. digestive system, other | 1.587 |
| K76 | Ischaemic heart dis. without angina | D99 | Dis. digestive system, other | 1.534 |
| T99 | Endocrine/metabolic/nutrition dis. other | D99 | Dis. digestive system, other | 1.53 |
| K87 | Hypertension complicated | D99 | Dis. digestive system, other | 1.433 |
| K78 | Atrial Fibrillation/flutter | D99 | Dis. digestive system, other | 1.41 |
| F92 | Cataract | D99 | Dis. digestive system, other | 1.383 |
| F84 | Macular degeneration | D99 | Dis. digestive system, other | 1.376 |
| P71 | Psychosis organic, other | D99 | Dis. digestive system, other | 1.359 |
| K89 | Transient cerebral ischaemia | D99 | Dis. digestive system, other | 1.35 |
| K84 | Heart dis. other | D99 | Dis. digestive system, other | 1.251 |
| T89 | Type 1 Diabetes mellitus | F83 | Retinopathy | 25.336 |
| B82 | Anaemia other, unspecified | F83 | Retinopathy | 2.34 |
| F84 | Macular degeneration | F83 | Retinopathy | 2.32 |
| K74 | Ischaemic heart dis. with angina | F83 | Retinopathy | 1.996 |
| B81 | Anaemia vitamin B12/folate deficiency | F83 | Retinopathy | 1.799 |
| K82 | Pulmonary heart dis. | F83 | Retinopathy | 1.79 |
| K89 | Transient cerebral ischaemia | F83 | Retinopathy | 1.567 |
| D98 | Cholecystitis/cholelithiasis | F83 | Retinopathy | 1.33 |
| X77 | Female genital ca, other | F83 | Retinopathy | 1.26 |
| F84 | Macular degeneration | F92 | Cataract | 2.199 |
| F83 | Retinopathy | F92 | Cataract | 1.997 |
| T89 | Type 1 Diabetes mellitus | F92 | Cataract | 1.467 |
| U99.01 | Chronic renal failure | F92 | Cataract | 1.428 |
| K87 | Hypertension complicated | F92 | Cataract | 1.415 |
| P71 | Psychosis organic, other | F92 | Cataract | 1.41 |
| K92 | Atherosclerosis/peripheral vascular dis | F92 | Cataract | 1.408 |
| K82 | Pulmonary heart dis. | F92 | Cataract | 1.407 |
| B82 | Anaemia other, unspecified | F92 | Cataract | 1.398 |
| D98 | Cholecystitis/cholelithiasis | F92 | Cataract | 1.39 |
| T90 | Type 2 Diabetes mellitus | F92 | Cataract | 1.379 |
| K74 | Ischaemic heart dis. with angina | F92 | Cataract | 1.37 |
| K89 | Transient cerebral ischaemia | F92 | Cataract | 1.323 |
| K91 | Cerebrovascular dis. | F92 | Cataract | 1.323 |
| B81 | Anaemia vitamin B12/folate deficiency | F92 | Cataract | 1.277 |
| T99 | Endocrine/metabolic/nutrition dis. other | F92 | Cataract | 1.273 |
| D97 | Liver dis. NOS | F92 | Cataract | 1.263 |
| K76 | Ischaemic heart dis. without angina | F92 | Cataract | 1.23 |
| T89 | Type 1 Diabetes mellitus | F93 | Glaucoma | 1.912 |
| F92 | Cataract | F93 | Glaucoma | 1.899 |
| F83 | Retinopathy | F93 | Glaucoma | 1.829 |
| F84 | Macular degeneration | F93 | Glaucoma | 1.687 |
| T90 | Type 2 Diabetes mellitus | F93 | Glaucoma | 1.506 |
| K92 | Atherosclerosis/peripheral vascular dis | F93 | Glaucoma | 1.254 |
| K91 | Cerebrovascular dis. | F93 | Glaucoma | 1.251 |
| K87 | Hypertension complicated | F93 | Glaucoma | 1.239 |
| K82 | Pulmonary heart dis. | K74 | Ischaemic heart dis. with angina | 1.856 |
| T89 | Type 1 Diabetes mellitus | K74 | Ischaemic heart dis. with angina | 1.747 |
| B82 | Anaemia other, unspecified | K74 | Ischaemic heart dis. with angina | 1.633 |
| D98 | Cholecystitis/cholelithiasis | K74 | Ischaemic heart dis. with angina | 1.413 |
| B81 | Anaemia vitamin B12/folate deficiency | K74 | Ischaemic heart dis. with angina | 1.294 |
| F84 | Macular degeneration | K74 | Ischaemic heart dis. with angina | 1.289 |
| T99 | Endocrine/metabolic/nutrition dis. other | K74 | Ischaemic heart dis. with angina | 1.279 |
| K76 | Ischaemic heart dis. without angina | K75 | Acute myocardial infarction | 11.301 |
| K74 | Ischaemic heart dis. with angina | K75 | Acute myocardial infarction | 5.429 |
| K77 | Heart failure | K75 | Acute myocardial infarction | 3.272 |
| T89 | Type 1 Diabetes mellitus | K75 | Acute myocardial infarction | 2.393 |
| K92 | Atherosclerosis/peripheral vascular dis | K75 | Acute myocardial infarction | 2.353 |
| F83 | Retinopathy | K75 | Acute myocardial infarction | 2.183 |
| T90 | Type 2 Diabetes mellitus | K75 | Acute myocardial infarction | 2.066 |
| U99.01 | Chronic renal failure | K75 | Acute myocardial infarction | 1.932 |
| T93 | Lipid disorder | K75 | Acute myocardial infarction | 1.728 |
| K78 | Atrial Fibrillation/flutter | K75 | Acute myocardial infarction | 1.72 |
| U88 | Glomerulonephritis/nephrosis | K75 | Acute myocardial infarction | 1.717 |
| K82 | Pulmonary heart dis. | K75 | Acute myocardial infarction | 1.696 |
| B82 | Anaemia other, unspecified | K75 | Acute myocardial infarction | 1.615 |
| K91 | Cerebrovascular dis. | K75 | Acute myocardial infarction | 1.519 |
| K86 | Hypertension uncomplicated | K75 | Acute myocardial infarction | 1.506 |
| D99 | Dis. digestive system, other | K75 | Acute myocardial infarction | 1.428 |
| K84 | Heart dis. other | K75 | Acute myocardial infarction | 1.403 |
| K89 | Transient cerebral ischaemia | K75 | Acute myocardial infarction | 1.318 |
| T83/82 | Obesity/overweight | K75 | Acute myocardial infarction | 1.264 |
| D98 | Cholecystitis/cholelithiasis | K75 | Acute myocardial infarction | 1.25 |
| K87 | Hypertension complicated | K75 | Acute myocardial infarction | 1.206 |
| T92 | Gout | K75 | Acute myocardial infarction | 1.204 |
| K74 | Ischaemic heart dis. with angina | K76 | Ischaemic heart dis. without angina | 7.594 |
| F83 | Retinopathy | K76 | Ischaemic heart dis. without angina | 2.459 |
| T89 | Type 1 Diabetes mellitus | K76 | Ischaemic heart dis. without angina | 2.439 |
| K82 | Pulmonary heart dis. | K76 | Ischaemic heart dis. without angina | 2.169 |
| B82 | Anaemia other, unspecified | K76 | Ischaemic heart dis. without angina | 1.748 |
| K89 | Transient cerebral ischaemia | K76 | Ischaemic heart dis. without angina | 1.399 |
| D98 | Cholecystitis/cholelithiasis | K76 | Ischaemic heart dis. without angina | 1.313 |
| T99 | Endocrine/metabolic/nutrition dis. other | K76 | Ischaemic heart dis. without angina | 1.302 |
| B81 | Anaemia vitamin B12/folate deficiency | K76 | Ischaemic heart dis. without angina | 1.229 |
| F84 | Macular degeneration | K76 | Ischaemic heart dis. without angina | 1.203 |
| K82 | Pulmonary heart dis. | K77 | Heart failure | 9.551 |
| K76 | Ischaemic heart dis. without angina | K77 | Heart failure | 3.42 |
| U99.01 | Chronic renal failure | K77 | Heart failure | 2.561 |
| K74 | Ischaemic heart dis. with angina | K77 | Heart failure | 2.554 |
| F83 | Retinopathy | K77 | Heart failure | 2.173 |
| T89 | Type 1 Diabetes mellitus | K77 | Heart failure | 2.117 |
| B82 | Anaemia other, unspecified | K77 | Heart failure | 2.1 |
| K92 | Atherosclerosis/peripheral vascular dis | K77 | Heart failure | 1.924 |
| T99 | Endocrine/metabolic/nutrition dis. other | K77 | Heart failure | 1.773 |
| B81 | Anaemia vitamin B12/folate deficiency | K77 | Heart failure | 1.381 |
| D98 | Cholecystitis/cholelithiasis | K77 | Heart failure | 1.368 |
| K89 | Transient cerebral ischaemia | K77 | Heart failure | 1.357 |
| K82 | Pulmonary heart dis. | K78 | Atrial Fibrillation/flutter | 8.088 |
| K77 | Heart failure | K78 | Atrial Fibrillation/flutter | 6.206 |
| K87 | Hypertension complicated | K78 | Atrial Fibrillation/flutter | 2.397 |
| K76 | Ischaemic heart dis. without angina | K78 | Atrial Fibrillation/flutter | 2.024 |
| K74 | Ischaemic heart dis. with angina | K78 | Atrial Fibrillation/flutter | 1.957 |
| U99.01 | Chronic renal failure | K78 | Atrial Fibrillation/flutter | 1.881 |
| T92 | Gout | K78 | Atrial Fibrillation/flutter | 1.775 |
| K84 | Heart dis. other | K78 | Atrial Fibrillation/flutter | 1.748 |
| K86 | Hypertension uncomplicated | K78 | Atrial Fibrillation/flutter | 1.737 |
| B82 | Anaemia other, unspecified | K78 | Atrial Fibrillation/flutter | 1.638 |
| K89 | Transient cerebral ischaemia | K78 | Atrial Fibrillation/flutter | 1.619 |
| K92 | Atherosclerosis/peripheral vascular dis | K78 | Atrial Fibrillation/flutter | 1.583 |
| T99 | Endocrine/metabolic/nutrition dis. other | K78 | Atrial Fibrillation/flutter | 1.541 |
| T83/82 | Obesity/overweight | K78 | Atrial Fibrillation/flutter | 1.54 |
| T90 | Type 2 Diabetes mellitus | K78 | Atrial Fibrillation/flutter | 1.372 |
| K91 | Cerebrovascular dis. | K78 | Atrial Fibrillation/flutter | 1.37 |
| D98 | Cholecystitis/cholelithiasis | K78 | Atrial Fibrillation/flutter | 1.351 |
| F83 | Retinopathy | K78 | Atrial Fibrillation/flutter | 1.35 |
| P71 | Psychosis organic, other | K78 | Atrial Fibrillation/flutter | 1.292 |
| F92 | Cataract | K78 | Atrial Fibrillation/flutter | 1.239 |
| K87 | Hypertension complicated | K84 | Heart dis. other | 2.8 |
| K82 | Pulmonary heart dis. | K84 | Heart dis. other | 2.583 |
| K77 | Heart failure | K84 | Heart dis. other | 2.404 |
| F83 | Retinopathy | K84 | Heart dis. other | 1.901 |
| U99.01 | Chronic renal failure | K84 | Heart dis. other | 1.784 |
| K76 | Ischaemic heart dis. without angina | K84 | Heart dis. other | 1.671 |
| K74 | Ischaemic heart dis. with angina | K84 | Heart dis. other | 1.654 |
| T90 | Type 2 Diabetes mellitus | K84 | Heart dis. other | 1.613 |
| T99 | Endocrine/metabolic/nutrition dis. other | K84 | Heart dis. other | 1.49 |
| F92 | Cataract | K84 | Heart dis. other | 1.483 |
| B82 | Anaemia other, unspecified | K84 | Heart dis. other | 1.466 |
| F84 | Macular degeneration | K84 | Heart dis. other | 1.421 |
| K92 | Atherosclerosis/peripheral vascular dis | K84 | Heart dis. other | 1.384 |
| D98 | Cholecystitis/cholelithiasis | K84 | Heart dis. other | 1.305 |
| K91 | Cerebrovascular dis. | K84 | Heart dis. other | 1.285 |
| K89 | Transient cerebral ischaemia | K84 | Heart dis. other | 1.264 |
| P71 | Psychosis organic, other | K84 | Heart dis. other | 1.239 |
| F93 | Glaucoma | K84 | Heart dis. other | 1.214 |
| K87 | Hypertension complicated | K86 | Hypertension uncomplicated | 9.578 |
| U99.01 | Chronic renal failure | K86 | Hypertension uncomplicated | 3.456 |
| F83 | Retinopathy | K86 | Hypertension uncomplicated | 3.352 |
| T90 | Type 2 Diabetes mellitus | K86 | Hypertension uncomplicated | 2.759 |
| K84 | Heart dis. other | K86 | Hypertension uncomplicated | 2.14 |
| T99 | Endocrine/metabolic/nutrition dis. other | K86 | Hypertension uncomplicated | 1.985 |
| K74 | Ischaemic heart dis. with angina | K86 | Hypertension uncomplicated | 1.9 |
| K91 | Cerebrovascular dis. | K86 | Hypertension uncomplicated | 1.891 |
| K76 | Ischaemic heart dis. without angina | K86 | Hypertension uncomplicated | 1.782 |
| K77 | Heart failure | K86 | Hypertension uncomplicated | 1.774 |
| K92 | Atherosclerosis/peripheral vascular dis | K86 | Hypertension uncomplicated | 1.714 |
| K89 | Transient cerebral ischaemia | K86 | Hypertension uncomplicated | 1.687 |
| K82 | Pulmonary heart dis. | K86 | Hypertension uncomplicated | 1.608 |
| T89 | Type 1 Diabetes mellitus | K86 | Hypertension uncomplicated | 1.55 |
| D97 | Liver dis. NOS | K86 | Hypertension uncomplicated | 1.434 |
| F93 | Glaucoma | K86 | Hypertension uncomplicated | 1.427 |
| B82 | Anaemia other, unspecified | K86 | Hypertension uncomplicated | 1.421 |
| F92 | Cataract | K86 | Hypertension uncomplicated | 1.353 |
| F84 | Macular degeneration | K86 | Hypertension uncomplicated | 1.264 |
| D98 | Cholecystitis/cholelithiasis | K86 | Hypertension uncomplicated | 1.247 |
| K82 | Pulmonary heart dis. | K87 | Hypertension complicated | 4.234 |
| K77 | Heart failure | K87 | Hypertension complicated | 2.58 |
| U99.01 | Chronic renal failure | K87 | Hypertension complicated | 2.332 |
| F83 | Retinopathy | K87 | Hypertension complicated | 2.188 |
| T99 | Endocrine/metabolic/nutrition dis. other | K87 | Hypertension complicated | 1.967 |
| K74 | Ischaemic heart dis. with angina | K87 | Hypertension complicated | 1.871 |
| K76 | Ischaemic heart dis. without angina | K87 | Hypertension complicated | 1.74 |
| B82 | Anaemia other, unspecified | K87 | Hypertension complicated | 1.659 |
| K92 | Atherosclerosis/peripheral vascular dis | K87 | Hypertension complicated | 1.657 |
| K89 | Transient cerebral ischaemia | K87 | Hypertension complicated | 1.465 |
| F84 | Macular degeneration | K87 | Hypertension complicated | 1.387 |
| D98 | Cholecystitis/cholelithiasis | K87 | Hypertension complicated | 1.352 |
| P71 | Psychosis organic, other | K87 | Hypertension complicated | 1.315 |
| T89 | Type 1 Diabetes mellitus | K87 | Hypertension complicated | 1.282 |
| B81 | Anaemia vitamin B12/folate deficiency | K87 | Hypertension complicated | 1.258 |
| K74 | Ischaemic heart dis. with angina | K89 | Transient cerebral ischaemia | 1.428 |
| B82 | Anaemia other, unspecified | K89 | Transient cerebral ischaemia | 1.358 |
| F84 | Macular degeneration | K89 | Transient cerebral ischaemia | 1.352 |
| B81 | Anaemia vitamin B12/folate deficiency | K89 | Transient cerebral ischaemia | 1.299 |
| T89 | Type 1 Diabetes mellitus | K89 | Transient cerebral ischaemia | 1.265 |
| D98 | Cholecystitis/cholelithiasis | K89 | Transient cerebral ischaemia | 1.23 |
| K91 | Cerebrovascular dis. | K90 | Stroke | 5.723 |
| K89 | Transient cerebral ischaemia | K90 | Stroke | 2.399 |
| K78 | Atrial Fibrillation/flutter | K90 | Stroke | 2.177 |
| F83 | Retinopathy | K90 | Stroke | 2.031 |
| K92 | Atherosclerosis/peripheral vascular dis | K90 | Stroke | 1.951 |
| K86 | Hypertension uncomplicated | K90 | Stroke | 1.937 |
| T89 | Type 1 Diabetes mellitus | K90 | Stroke | 1.922 |
| P71 | Psychosis organic, other | K90 | Stroke | 1.71 |
| T90 | Type 2 Diabetes mellitus | K90 | Stroke | 1.644 |
| K77 | Heart failure | K90 | Stroke | 1.594 |
| K75 | Acute myocardial infarction | K90 | Stroke | 1.574 |
| K76 | Ischaemic heart dis. without angina | K90 | Stroke | 1.564 |
| N94 | Peripheral neuritis/neuropathy | K90 | Stroke | 1.539 |
| K87 | Hypertension complicated | K90 | Stroke | 1.506 |
| U88 | Glomerulonephritis/nephrosis | K90 | Stroke | 1.484 |
| U99.01 | Chronic renal failure | K90 | Stroke | 1.46 |
| B82 | Anaemia other, unspecified | K90 | Stroke | 1.446 |
| B81 | Anaemia vitamin B12/folate deficiency | K90 | Stroke | 1.438 |
| D99 | Dis. digestive system, other | K90 | Stroke | 1.432 |
| K74 | Ischaemic heart dis. with angina | K90 | Stroke | 1.357 |
| K82 | Pulmonary heart dis. | K90 | Stroke | 1.227 |
| T93 | Lipid disorder | K90 | Stroke | 1.204 |
| K89 | Transient cerebral ischaemia | K91 | Cerebrovascular dis. | 3.717 |
| K92 | Atherosclerosis/peripheral vascular dis | K91 | Cerebrovascular dis. | 3.098 |
| P71 | Psychosis organic, other | K91 | Cerebrovascular dis. | 1.949 |
| F83 | Retinopathy | K91 | Cerebrovascular dis. | 1.879 |
| K76 | Ischaemic heart dis. without angina | K91 | Cerebrovascular dis. | 1.847 |
| K87 | Hypertension complicated | K91 | Cerebrovascular dis. | 1.815 |
| T89 | Type 1 Diabetes mellitus | K91 | Cerebrovascular dis. | 1.604 |
| K74 | Ischaemic heart dis. with angina | K91 | Cerebrovascular dis. | 1.568 |
| B82 | Anaemia other, unspecified | K91 | Cerebrovascular dis. | 1.565 |
| U99.01 | Chronic renal failure | K91 | Cerebrovascular dis. | 1.55 |
| F84 | Macular degeneration | K91 | Cerebrovascular dis. | 1.406 |
| B81 | Anaemia vitamin B12/folate deficiency | K91 | Cerebrovascular dis. | 1.379 |
| K77 | Heart failure | K91 | Cerebrovascular dis. | 1.36 |
| D98 | Cholecystitis/cholelithiasis | K91 | Cerebrovascular dis. | 1.247 |
| T99 | Endocrine/metabolic/nutrition dis. other | K91 | Cerebrovascular dis. | 1.201 |
| F83 | Retinopathy | K92 | Atherosclerosis/peripheral vascular dis | 3.212 |
| T89 | Type 1 Diabetes mellitus | K92 | Atherosclerosis/peripheral vascular dis | 3.053 |
| K76 | Ischaemic heart dis. without angina | K92 | Atherosclerosis/peripheral vascular dis | 2.464 |
| K74 | Ischaemic heart dis. with angina | K92 | Atherosclerosis/peripheral vascular dis | 2.059 |
| K82 | Pulmonary heart dis. | K92 | Atherosclerosis/peripheral vascular dis | 1.893 |
| B82 | Anaemia other, unspecified | K92 | Atherosclerosis/peripheral vascular dis | 1.857 |
| K89 | Transient cerebral ischaemia | K92 | Atherosclerosis/peripheral vascular dis | 1.739 |
| B81 | Anaemia vitamin B12/folate deficiency | K92 | Atherosclerosis/peripheral vascular dis | 1.406 |
| F84 | Macular degeneration | K92 | Atherosclerosis/peripheral vascular dis | 1.354 |
| T99 | Endocrine/metabolic/nutrition dis. other | K92 | Atherosclerosis/peripheral vascular dis | 1.318 |
| D98 | Cholecystitis/cholelithiasis | K92 | Atherosclerosis/peripheral vascular dis | 1.261 |
| D77 | Digestive ca. other/NOS | K92 | Atherosclerosis/peripheral vascular dis | 1.235 |
| N94 | Peripheral neuritis/neuropathy | N93 | Carpal tunnel syndrome | 2.549 |
| T83/82 | Obesity/overweight | N93 | Carpal tunnel syndrome | 1.588 |
| F83 | Retinopathy | N93 | Carpal tunnel syndrome | 1.53 |
| T89 | Type 1 Diabetes mellitus | N93 | Carpal tunnel syndrome | 1.464 |
| F92 | Cataract | N93 | Carpal tunnel syndrome | 1.384 |
| D97 | Liver dis. NOS | N93 | Carpal tunnel syndrome | 1.38 |
| T92 | Gout | N93 | Carpal tunnel syndrome | 1.319 |
| F84 | Macular degeneration | N93 | Carpal tunnel syndrome | 1.29 |
| K74 | Ischaemic heart dis. with angina | N93 | Carpal tunnel syndrome | 1.29 |
| D98 | Cholecystitis/cholelithiasis | N93 | Carpal tunnel syndrome | 1.26 |
| K92 | Atherosclerosis/peripheral vascular dis | N93 | Carpal tunnel syndrome | 1.259 |
| T93 | Lipid disorder | N93 | Carpal tunnel syndrome | 1.256 |
| K87 | Hypertension complicated | N93 | Carpal tunnel syndrome | 1.246 |
| T90 | Type 2 Diabetes mellitus | N93 | Carpal tunnel syndrome | 1.24 |
| F93 | Glaucoma | N93 | Carpal tunnel syndrome | 1.224 |
| F83 | Retinopathy | N94 | Peripheral neuritis/neuropathy | 5.704 |
| T89 | Type 1 Diabetes mellitus | N94 | Peripheral neuritis/neuropathy | 5.224 |
| K92 | Atherosclerosis/peripheral vascular dis | N94 | Peripheral neuritis/neuropathy | 2.752 |
| U88 | Glomerulonephritis/nephrosis | N94 | Peripheral neuritis/neuropathy | 2.713 |
| T90 | Type 2 Diabetes mellitus | N94 | Peripheral neuritis/neuropathy | 2.704 |
| D99 | Dis. digestive system, other | N94 | Peripheral neuritis/neuropathy | 1.976 |
| B81 | Anaemia vitamin B12/folate deficiency | N94 | Peripheral neuritis/neuropathy | 1.887 |
| K91 | Cerebrovascular dis. | N94 | Peripheral neuritis/neuropathy | 1.737 |
| D97 | Liver dis. NOS | N94 | Peripheral neuritis/neuropathy | 1.708 |
| K76 | Ischaemic heart dis. without angina | N94 | Peripheral neuritis/neuropathy | 1.683 |
| B82 | Anaemia other, unspecified | N94 | Peripheral neuritis/neuropathy | 1.682 |
| K77 | Heart failure | N94 | Peripheral neuritis/neuropathy | 1.644 |
| K74 | Ischaemic heart dis. with angina | N94 | Peripheral neuritis/neuropathy | 1.639 |
| U99.01 | Chronic renal failure | N94 | Peripheral neuritis/neuropathy | 1.619 |
| F92 | Cataract | N94 | Peripheral neuritis/neuropathy | 1.565 |
| K82 | Pulmonary heart dis. | N94 | Peripheral neuritis/neuropathy | 1.531 |
| F84 | Macular degeneration | N94 | Peripheral neuritis/neuropathy | 1.479 |
| K75 | Acute myocardial infarction | N94 | Peripheral neuritis/neuropathy | 1.479 |
| K87 | Hypertension complicated | N94 | Peripheral neuritis/neuropathy | 1.473 |
| K89 | Transient cerebral ischaemia | N94 | Peripheral neuritis/neuropathy | 1.438 |
| F93 | Glaucoma | N94 | Peripheral neuritis/neuropathy | 1.391 |
| T83/82 | Obesity/overweight | N94 | Peripheral neuritis/neuropathy | 1.39 |
| P71 | Psychosis organic, other | N94 | Peripheral neuritis/neuropathy | 1.388 |
| K84 | Heart dis. other | N94 | Peripheral neuritis/neuropathy | 1.372 |
| D98 | Cholecystitis/cholelithiasis | N94 | Peripheral neuritis/neuropathy | 1.353 |
| K86 | Hypertension uncomplicated | N94 | Peripheral neuritis/neuropathy | 1.301 |
| D77 | Digestive ca. other/NOS | N94 | Peripheral neuritis/neuropathy | 1.291 |
| X77 | Female genital ca, other | N94 | Peripheral neuritis/neuropathy | 1.224 |
| T99 | Endocrine/metabolic/nutrition dis. other | N94 | Peripheral neuritis/neuropathy | 1.206 |
| B81 | Anaemia vitamin B12/folate deficiency | P71 | Psychosis organic, other | 1.671 |
| B82 | Anaemia other, unspecified | P71 | Psychosis organic, other | 1.584 |
| K89 | Transient cerebral ischaemia | P71 | Psychosis organic, other | 1.56 |
| F83 | Retinopathy | P71 | Psychosis organic, other | 1.436 |
| F84 | Macular degeneration | P71 | Psychosis organic, other | 1.429 |
| U99.01 | Chronic renal failure | P71 | Psychosis organic, other | 1.399 |
| K92 | Atherosclerosis/peripheral vascular dis | P71 | Psychosis organic, other | 1.294 |
| K77 | Heart failure | P71 | Psychosis organic, other | 1.225 |
| K76 | Ischaemic heart dis. without angina | P71 | Psychosis organic, other | 1.217 |
| P71 | Psychosis organic, other | P72 | Schizophrenia | 3.151 |
| T83/82 | Obesity/overweight | P72 | Schizophrenia | 1.448 |
| X77 | Female genital ca, other | P72 | Schizophrenia | 1.42 |
| T90 | Type 2 Diabetes mellitus | P72 | Schizophrenia | 1.293 |
| K86 | Hypertension uncomplicated | T83/82 | Obesity/overweight | 2.857 |
| T90 | Type 2 Diabetes mellitus | T83/82 | Obesity/overweight | 2.584 |
| K87 | Hypertension complicated | T83/82 | Obesity/overweight | 2.021 |
| F83 | Retinopathy | T83/82 | Obesity/overweight | 2.003 |
| K82 | Pulmonary heart dis. | T83/82 | Obesity/overweight | 1.81 |
| D97 | Liver dis. NOS | T83/82 | Obesity/overweight | 1.792 |
| T99 | Endocrine/metabolic/nutrition dis. other | T83/82 | Obesity/overweight | 1.679 |
| K77 | Heart failure | T83/82 | Obesity/overweight | 1.677 |
| U99.01 | Chronic renal failure | T83/82 | Obesity/overweight | 1.579 |
| D98 | Cholecystitis/cholelithiasis | T83/82 | Obesity/overweight | 1.55 |
| K84 | Heart dis. other | T83/82 | Obesity/overweight | 1.51 |
| K74 | Ischaemic heart dis. with angina | T83/82 | Obesity/overweight | 1.458 |
| K76 | Ischaemic heart dis. without angina | T83/82 | Obesity/overweight | 1.36 |
| X77 | Female genital ca, other | T83/82 | Obesity/overweight | 1.319 |
| F92 | Cataract | T83/82 | Obesity/overweight | 1.277 |
| D76 | Pancreas Ca. | T89 | Type 1 Diabetes mellitus | 4.01 |
| B81 | Anaemia vitamin B12/folate deficiency | T89 | Type 1 Diabetes mellitus | 1.884 |
| F83 | Retinopathy | T90 | Type 2 Diabetes mellitus | 23.829 |
| T89 | Type 1 Diabetes mellitus | T90 | Type 2 Diabetes mellitus | 3.925 |
| D76 | Pancreas Ca. | T90 | Type 2 Diabetes mellitus | 2.423 |
| K76 | Ischaemic heart dis. without angina | T90 | Type 2 Diabetes mellitus | 2.161 |
| K92 | Atherosclerosis/peripheral vascular dis | T90 | Type 2 Diabetes mellitus | 2.039 |
| K74 | Ischaemic heart dis. with angina | T90 | Type 2 Diabetes mellitus | 1.894 |
| D97 | Liver dis. NOS | T90 | Type 2 Diabetes mellitus | 1.89 |
| K77 | Heart failure | T90 | Type 2 Diabetes mellitus | 1.867 |
| U99.01 | Chronic renal failure | T90 | Type 2 Diabetes mellitus | 1.763 |
| B82 | Anaemia other, unspecified | T90 | Type 2 Diabetes mellitus | 1.725 |
| K87 | Hypertension complicated | T90 | Type 2 Diabetes mellitus | 1.692 |
| K82 | Pulmonary heart dis. | T90 | Type 2 Diabetes mellitus | 1.647 |
| B81 | Anaemia vitamin B12/folate deficiency | T90 | Type 2 Diabetes mellitus | 1.438 |
| K91 | Cerebrovascular dis. | T90 | Type 2 Diabetes mellitus | 1.416 |
| X77 | Female genital ca, other | T90 | Type 2 Diabetes mellitus | 1.38 |
| D98 | Cholecystitis/cholelithiasis | T90 | Type 2 Diabetes mellitus | 1.341 |
| T99 | Endocrine/metabolic/nutrition dis. other | T90 | Type 2 Diabetes mellitus | 1.31 |
| F84 | Macular degeneration | T90 | Type 2 Diabetes mellitus | 1.26 |
| K89 | Transient cerebral ischaemia | T90 | Type 2 Diabetes mellitus | 1.246 |
| D77 | Digestive ca. other/NOS | T90 | Type 2 Diabetes mellitus | 1.222 |
| P71 | Psychosis organic, other | T90 | Type 2 Diabetes mellitus | 1.209 |
| B78 | Hereditary haemolytic anaemia | T90 | Type 2 Diabetes mellitus | 1.201 |
| T99 | Endocrine/metabolic/nutrition dis. other | T92 | Gout | 5.105 |
| U99.01 | Chronic renal failure | T92 | Gout | 3.254 |
| K86 | Hypertension uncomplicated | T92 | Gout | 2.43 |
| K82 | Pulmonary heart dis. | T92 | Gout | 2.056 |
| T83/82 | Obesity/overweight | T92 | Gout | 2.004 |
| K77 | Heart failure | T92 | Gout | 1.922 |
| K87 | Hypertension complicated | T92 | Gout | 1.818 |
| B82 | Anaemia other, unspecified | T92 | Gout | 1.622 |
| D97 | Liver dis. NOS | T92 | Gout | 1.508 |
| K84 | Heart dis. other | T92 | Gout | 1.45 |
| T90 | Type 2 Diabetes mellitus | T92 | Gout | 1.346 |
| X77 | Female genital ca, other | T92 | Gout | 1.342 |
| K74 | Ischaemic heart dis. with angina | T92 | Gout | 1.316 |
| F92 | Cataract | T92 | Gout | 1.297 |
| K76 | Ischaemic heart dis. without angina | T92 | Gout | 1.293 |
| K92 | Atherosclerosis/peripheral vascular dis | T92 | Gout | 1.278 |
| D98 | Cholecystitis/cholelithiasis | T92 | Gout | 1.274 |
| K89 | Transient cerebral ischaemia | T92 | Gout | 1.243 |
| K76 | Ischaemic heart dis. without angina | T93 | Lipid disorder | 1.808 |
| K74 | Ischaemic heart dis. with angina | T93 | Lipid disorder | 1.789 |
| T90 | Type 2 Diabetes mellitus | T93 | Lipid disorder | 1.691 |
| K86 | Hypertension uncomplicated | T93 | Lipid disorder | 1.686 |
| T92 | Gout | T93 | Lipid disorder | 1.566 |
| F83 | Retinopathy | T93 | Lipid disorder | 1.476 |
| U99.01 | Chronic renal failure | T93 | Lipid disorder | 1.463 |
| K91 | Cerebrovascular dis. | T93 | Lipid disorder | 1.459 |
| K87 | Hypertension complicated | T93 | Lipid disorder | 1.444 |
| T99 | Endocrine/metabolic/nutrition dis. other | T93 | Lipid disorder | 1.44 |
| K92 | Atherosclerosis/peripheral vascular dis | T93 | Lipid disorder | 1.42 |
| T83/82 | Obesity/overweight | T93 | Lipid disorder | 1.402 |
| D97 | Liver dis. NOS | T93 | Lipid disorder | 1.353 |
| K89 | Transient cerebral ischaemia | T93 | Lipid disorder | 1.311 |
| F92 | Cataract | T93 | Lipid disorder | 1.265 |
| K84 | Heart dis. other | T93 | Lipid disorder | 1.253 |
| F93 | Glaucoma | T93 | Lipid disorder | 1.227 |
| K82 | Pulmonary heart dis. | T99 | Endocrine/metabolic/nutrition dis. other | 2.05 |
| B82 | Anaemia other, unspecified | T99 | Endocrine/metabolic/nutrition dis. other | 1.599 |
| B81 | Anaemia vitamin B12/folate deficiency | T99 | Endocrine/metabolic/nutrition dis. other | 1.362 |
| F84 | Macular degeneration | T99 | Endocrine/metabolic/nutrition dis. other | 1.205 |
| T89 | Type 1 Diabetes mellitus | U88 | Glomerulonephritis/nephrosis | 7.333 |
| F83 | Retinopathy | U88 | Glomerulonephritis/nephrosis | 5.951 |
| T90 | Type 2 Diabetes mellitus | U88 | Glomerulonephritis/nephrosis | 3.443 |
| K86 | Hypertension uncomplicated | U88 | Glomerulonephritis/nephrosis | 3.021 |
| U99.01 | Chronic renal failure | U88 | Glomerulonephritis/nephrosis | 2.421 |
| K92 | Atherosclerosis/peripheral vascular dis | U88 | Glomerulonephritis/nephrosis | 2.188 |
| K87 | Hypertension complicated | U88 | Glomerulonephritis/nephrosis | 2.139 |
| B82 | Anaemia other, unspecified | U88 | Glomerulonephritis/nephrosis | 2.119 |
| T92 | Gout | U88 | Glomerulonephritis/nephrosis | 1.931 |
| T99 | Endocrine/metabolic/nutrition dis. other | U88 | Glomerulonephritis/nephrosis | 1.929 |
| K76 | Ischaemic heart dis. without angina | U88 | Glomerulonephritis/nephrosis | 1.893 |
| K82 | Pulmonary heart dis. | U88 | Glomerulonephritis/nephrosis | 1.833 |
| K77 | Heart failure | U88 | Glomerulonephritis/nephrosis | 1.727 |
| K91 | Cerebrovascular dis. | U88 | Glomerulonephritis/nephrosis | 1.625 |
| K74 | Ischaemic heart dis. with angina | U88 | Glomerulonephritis/nephrosis | 1.603 |
| T83/82 | Obesity/overweight | U88 | Glomerulonephritis/nephrosis | 1.559 |
| K84 | Heart dis. other | U88 | Glomerulonephritis/nephrosis | 1.532 |
| T93 | Lipid disorder | U88 | Glomerulonephritis/nephrosis | 1.52 |
| F92 | Cataract | U88 | Glomerulonephritis/nephrosis | 1.457 |
| K78 | Atrial Fibrillation/flutter | U88 | Glomerulonephritis/nephrosis | 1.428 |
| B81 | Anaemia vitamin B12/folate deficiency | U88 | Glomerulonephritis/nephrosis | 1.425 |
| K89 | Transient cerebral ischaemia | U88 | Glomerulonephritis/nephrosis | 1.346 |
| F93 | Glaucoma | U88 | Glomerulonephritis/nephrosis | 1.334 |
| D98 | Cholecystitis/cholelithiasis | U88 | Glomerulonephritis/nephrosis | 1.301 |
| D97 | Liver dis. NOS | U88 | Glomerulonephritis/nephrosis | 1.282 |
| P71 | Psychosis organic, other | U88 | Glomerulonephritis/nephrosis | 1.28 |
| T99 | Endocrine/metabolic/nutrition dis. other | U99.01 | Chronic renal failure | 3.233 |
| B82 | Anaemia other, unspecified | U99.01 | Chronic renal failure | 3.055 |
| F83 | Retinopathy | U99.01 | Chronic renal failure | 2.695 |
| K82 | Pulmonary heart dis. | U99.01 | Chronic renal failure | 2.569 |
| T89 | Type 1 Diabetes mellitus | U99.01 | Chronic renal failure | 2.151 |
| K92 | Atherosclerosis/peripheral vascular dis | U99.01 | Chronic renal failure | 2.094 |
| K76 | Ischaemic heart dis. without angina | U99.01 | Chronic renal failure | 1.964 |
| K74 | Ischaemic heart dis. with angina | U99.01 | Chronic renal failure | 1.771 |
| B81 | Anaemia vitamin B12/folate deficiency | U99.01 | Chronic renal failure | 1.583 |
| K89 | Transient cerebral ischaemia | U99.01 | Chronic renal failure | 1.402 |
| D98 | Cholecystitis/cholelithiasis | U99.01 | Chronic renal failure | 1.353 |
| F84 | Macular degeneration | U99.01 | Chronic renal failure | 1.34 |
| D77 | Digestive ca. other/NOS | X77 | Female genital ca, other | 1.879 |
| D76 | Pancreas Ca. | X77 | Female genital ca, other | 1.857 |

**Table S6. Degree and PageRank of T2DM nodes in the global multimorbidity network**

| Nodes in T90 subnet | | Global multimorbidity network | | | | Prevalence of disease |
| --- | --- | --- | --- | --- | --- | --- |
| ICPC2 code | Description | Degree | Position of Degree | PageRank | Position of PageRank |  |
| K82 | Pulmonary heart dis. | 59 | 37 | 0.0124 | 2 | 0.17% |
| N94 | Peripheral neuritis/neuropathy | 93 | 1 | 0.0119 | 4 | 1.25% |
| B82 | Anaemia other, unspecified | 89 | 2 | 0.0116 | 5 | 2.33% |
| D99 | Dis. digestive system, other | 76 | 9 | 0.0109 | 9 | 0.37% |
| P71 | Psychosis organic, other | 76 | 10 | 0.0101 | 14 | 1.11% |
| F83 | Retinopathy | 40 | 72 | 0.0099 | 19 | 0.94% |
| K92 | Atherosclerosis/peripheral vascular dis | 74 | 11 | 0.0096 | 21 | 1.69% |
| K77 | Heart failure | 58 | 40 | 0.0095 | 23 | 0.82% |
| D98 | Cholecystitis/cholelithiasis | 81 | 6 | 0.0094 | 24 | 2.60% |
| K91 | Cerebrovascular dis. | 70 | 14 | 0.0093 | 27 | 0.58% |
| F92 | Cataract | 80 | 7 | 0.0092 | 29 | 5.36% |
| K87 | Hypertension complicated | 63 | 28 | 0.0091 | 33 | 1.01% |
| U99.01 | Chronic renal failure | 62 | 32 | 0.0087 | 35 | 3.65% |
| B81 | Anaemia vitamin B12/folate deficiency | 64 | 23 | 0.0085 | 36 | 0.42% |
| D97 | Liver dis. NOS | 65 | 21 | 0.0083 | 39 | 3.36% |
| K74 | Ischaemic heart dis. with angina | 62 | 30 | 0.0083 | 40 | 0.71% |
| K89 | Transient cerebral ischaemia | 68 | 16 | 0.0080 | 44 | 0.53% |
| K78 | Atrial Fibrillation/flutter | 47 | 58 | 0.0079 | 47 | 2.38% |
| T99 | Endocrine/metabolic/nutrition dis. other | 58 | 42 | 0.0078 | 51 | 5.23% |
| F84 | Macular degeneration | 64 | 25 | 0.0076 | 52 | 0.49% |
| T89 | Type 1 Diabetes mellitus | 29 | 96 | 0.0074 | 58 | 0.30% |
| K84 | Heart dis. other | 57 | 44 | 0.0072 | 63 | 3.40% |
| T90 | Type 2 Diabetes mellitus | 37 | 80 | 0.0072 | 65 | 7.86% |
| K76 | Ischaemic heart dis. without angina | 44 | 68 | 0.0072 | 67 | 1.52% |
| U88 | Glomerulonephritis/nephrosis | 46 | 63 | 0.0071 | 68 | 0.42% |
| K90 | Stroke | 50 | 51 | 0.0071 | 69 | 1.29% |
| P72 | Schizophrenia | 27 | 102 | 0.0069 | 72 | 0.45% |
| D77 | Digestive ca. other/NOS | 32 | 91 | 0.0065 | 80 | 0.17% |
| K86 | Hypertension uncomplicated | 40 | 73 | 0.0065 | 81 | 21.10% |
| T92 | Gout | 46 | 62 | 0.0063 | 86 | 1.74% |
| N93 | Carpal tunnel syndrome | 47 | 61 | 0.0061 | 89 | 3.16% |
| K75 | Acute myocardial infarction | 35 | 84 | 0.0059 | 92 | 0.80% |
| T83/82 | Obesity/overweight | 40 | 75 | 0.0055 | 97 | 14.65% |
| X77 | Female genital ca, other | 24 | 113 | 0.0053 | 103 | 0.21% |
| F93 | Glaucoma | 33 | 88 | 0.0042 | 119 | 3.00% |
| D76 | Pancreas ca. | 14 | 132 | 0.0042 | 120 | 0.03% |
| T93 | Lipid disorder | 29 | 97 | 0.0039 | 128 | 21.19% |
| B78 | Hereditary haemolytic anaemia | 9 | 143 | 0.0020 | 147 | 0.37% |

T90: Type 2 diabetes mellitus. Dis: disease, Ca: cancer, NOS: not otherwise specified. Networks using ICPC-2 code system and OR> 1.2. Estimated prevalence of diseases in 2017

**Table S7. Odds ratio values for all nodes connected in directed diabetes network (for OR >1.2).**

| **from** |  | **to** |  | **OR** |
| --- | --- | --- | --- | --- |
| B82 | Anaemia other, unspecified | B78 | Hereditary haemolytic anaemia | 2.097 |
| T90 | Type 2 Diabetes mellitus | B78 | Hereditary haemolytic anaemia | 1.201 |
| B78 | Hereditary haemolytic anaemia | B81 | Anaemia vitamin B12/folate deficiency | 1.489 |
| B82 | Anaemia other, unspecified | B81 | Anaemia vitamin B12/folate deficiency | 2.101 |
| D77 | Digestive ca. other/NOS | B81 | Anaemia vitamin B12/folate deficiency | 1.243 |
| D98 | Cholecystitis/cholelithiasis | B81 | Anaemia vitamin B12/folate deficiency | 1.478 |
| F83 | Retinopathy | B81 | Anaemia vitamin B12/folate deficiency | 1.799 |
| K74 | Ischaemic heart dis. with angina | B81 | Anaemia vitamin B12/folate deficiency | 1.294 |
| K76 | Ischaemic heart dis. without angina | B81 | Anaemia vitamin B12/folate deficiency | 1.229 |
| K77 | Heart failure | B81 | Anaemia vitamin B12/folate deficiency | 1.381 |
| K82 | Pulmonary heart dis. | B81 | Anaemia vitamin B12/folate deficiency | 1.424 |
| K87 | Hypertension complicated | B81 | Anaemia vitamin B12/folate deficiency | 1.258 |
| K89 | Transient cerebral ischaemia | B81 | Anaemia vitamin B12/folate deficiency | 1.299 |
| K92 | Atherosclerosis/peripheral vascular dis | B81 | Anaemia vitamin B12/folate deficiency | 1.406 |
| P71 | Psychosis organic, other | B81 | Anaemia vitamin B12/folate deficiency | 1.671 |
| T89 | Type 1 Diabetes mellitus | B81 | Anaemia vitamin B12/folate deficiency | 1.884 |
| T90 | Type 2 Diabetes mellitus | B81 | Anaemia vitamin B12/folate deficiency | 1.438 |
| T99 | Endocrine/metabolic/nutrition dis. other | B81 | Anaemia vitamin B12/folate deficiency | 1.362 |
| U88 | Glomerulonephritis/nephrosis | B81 | Anaemia vitamin B12/folate deficiency | 1.425 |
| U99.01 | Chronic renal failure | B81 | Anaemia vitamin B12/folate deficiency | 1.583 |
| B78 | Hereditary haemolytic anaemia | B82 | Anaemia other, unspecified | 2.097 |
| D77 | Digestive ca. other/NOS | B82 | Anaemia other, unspecified | 1.481 |
| D98 | Cholecystitis/cholelithiasis | B82 | Anaemia other, unspecified | 1.32 |
| F83 | Retinopathy | B82 | Anaemia other, unspecified | 2.34 |
| F92 | Cataract | B82 | Anaemia other, unspecified | 1.398 |
| K74 | Ischaemic heart dis. with angina | B82 | Anaemia other, unspecified | 1.633 |
| K75 | Acute myocardial infarction | B82 | Anaemia other, unspecified | 1.615 |
| K76 | Ischaemic heart dis. without angina | B82 | Anaemia other, unspecified | 1.748 |
| K77 | Heart failure | B82 | Anaemia other, unspecified | 2.1 |
| K82 | Pulmonary heart dis. | B82 | Anaemia other, unspecified | 2.316 |
| K84 | Heart dis. other | B82 | Anaemia other, unspecified | 1.466 |
| K86 | Hypertension uncomplicated | B82 | Anaemia other, unspecified | 1.421 |
| K87 | Hypertension complicated | B82 | Anaemia other, unspecified | 1.659 |
| K89 | Transient cerebral ischaemia | B82 | Anaemia other, unspecified | 1.358 |
| K90 | Stroke | B82 | Anaemia other, unspecified | 1.446 |
| K91 | Cerebrovascular dis. | B82 | Anaemia other, unspecified | 1.565 |
| K92 | Atherosclerosis/peripheral vascular dis | B82 | Anaemia other, unspecified | 1.857 |
| N94 | Peripheral neuritis/neuropathy | B82 | Anaemia other, unspecified | 1.682 |
| P71 | Psychosis organic, other | B82 | Anaemia other, unspecified | 1.584 |
| T89 | Type 1 Diabetes mellitus | B82 | Anaemia other, unspecified | 1.397 |
| T90 | Type 2 Diabetes mellitus | B82 | Anaemia other, unspecified | 1.725 |
| T99 | Endocrine/metabolic/nutrition dis. other | B82 | Anaemia other, unspecified | 1.599 |
| U88 | Glomerulonephritis/nephrosis | B82 | Anaemia other, unspecified | 2.119 |
| U99.01 | Chronic renal failure | B82 | Anaemia other, unspecified | 3.055 |
| D77 | Digestive ca. other/NOS | D76 | Pancreas Ca. | 2.941 |
| D97 | Liver dis. NOS | D76 | Pancreas Ca. | 1.375 |
| D98 | Cholecystitis/cholelithiasis | D76 | Pancreas Ca. | 1.821 |
| T89 | Type 1 Diabetes mellitus | D76 | Pancreas Ca. | 4.01 |
| T90 | Type 2 Diabetes mellitus | D76 | Pancreas Ca. | 2.423 |
| X77 | Female genital ca, other | D76 | Pancreas Ca. | 1.857 |
| B81 | Anaemia vitamin B12/folate deficiency | D77 | Digestive ca. other/NOS | 1.243 |
| D76 | Pancreas Ca. | D77 | Digestive ca. other/NOS | 2.941 |
| D97 | Liver dis. NOS | D77 | Digestive ca. other/NOS | 3.989 |
| D98 | Cholecystitis/cholelithiasis | D77 | Digestive ca. other/NOS | 1.744 |
| D99 | Dis. digestive system, other | D77 | Digestive ca. other/NOS | 1.879 |
| K92 | Atherosclerosis/peripheral vascular dis | D77 | Digestive ca. other/NOS | 1.235 |
| N94 | Peripheral neuritis/neuropathy | D77 | Digestive ca. other/NOS | 1.291 |
| T90 | Type 2 Diabetes mellitus | D77 | Digestive ca. other/NOS | 1.222 |
| X77 | Female genital ca, other | D77 | Digestive ca. other/NOS | 1.879 |
| B81 | Anaemia vitamin B12/folate deficiency | D97 | Liver dis. NOS | 1.433 |
| B82 | Anaemia other, unspecified | D97 | Liver dis. NOS | 1.441 |
| D77 | Digestive ca. other/NOS | D97 | Liver dis. NOS | 3.989 |
| D98 | Cholecystitis/cholelithiasis | D97 | Liver dis. NOS | 2.945 |
| D99 | Dis. digestive system, other | D97 | Liver dis. NOS | 2.209 |
| F83 | Retinopathy | D97 | Liver dis. NOS | 1.307 |
| F92 | Cataract | D97 | Liver dis. NOS | 1.263 |
| K77 | Heart failure | D97 | Liver dis. NOS | 1.283 |
| K86 | Hypertension uncomplicated | D97 | Liver dis. NOS | 1.434 |
| K87 | Hypertension complicated | D97 | Liver dis. NOS | 1.545 |
| K92 | Atherosclerosis/peripheral vascular dis | D97 | Liver dis. NOS | 1.237 |
| N93 | Carpal tunnel syndrome | D97 | Liver dis. NOS | 1.38 |
| N94 | Peripheral neuritis/neuropathy | D97 | Liver dis. NOS | 1.708 |
| P71 | Psychosis organic, other | D97 | Liver dis. NOS | 1.2 |
| T83/82 | Obesity/overweight | D97 | Liver dis. NOS | 1.792 |
| T90 | Type 2 Diabetes mellitus | D97 | Liver dis. NOS | 1.89 |
| T92 | Gout | D97 | Liver dis. NOS | 1.508 |
| T93 | Lipid disorder | D97 | Liver dis. NOS | 1.353 |
| T99 | Endocrine/metabolic/nutrition dis. other | D97 | Liver dis. NOS | 1.577 |
| U88 | Glomerulonephritis/nephrosis | D97 | Liver dis. NOS | 1.282 |
| X77 | Female genital ca, other | D97 | Liver dis. NOS | 1.27 |
| B78 | Hereditary haemolytic anaemia | D98 | Cholecystitis/cholelithiasis | 1.245 |
| B81 | Anaemia vitamin B12/folate deficiency | D98 | Cholecystitis/cholelithiasis | 1.478 |
| B82 | Anaemia other, unspecified | D98 | Cholecystitis/cholelithiasis | 1.32 |
| D77 | Digestive ca. other/NOS | D98 | Cholecystitis/cholelithiasis | 1.744 |
| D99 | Dis. digestive system, other | D98 | Cholecystitis/cholelithiasis | 2.73 |
| F83 | Retinopathy | D98 | Cholecystitis/cholelithiasis | 1.33 |
| F92 | Cataract | D98 | Cholecystitis/cholelithiasis | 1.39 |
| K74 | Ischaemic heart dis. with angina | D98 | Cholecystitis/cholelithiasis | 1.413 |
| K75 | Acute myocardial infarction | D98 | Cholecystitis/cholelithiasis | 1.25 |
| K76 | Ischaemic heart dis. without angina | D98 | Cholecystitis/cholelithiasis | 1.313 |
| K77 | Heart failure | D98 | Cholecystitis/cholelithiasis | 1.368 |
| K78 | Atrial Fibrillation/flutter | D98 | Cholecystitis/cholelithiasis | 1.351 |
| K84 | Heart dis. other | D98 | Cholecystitis/cholelithiasis | 1.305 |
| K86 | Hypertension uncomplicated | D98 | Cholecystitis/cholelithiasis | 1.247 |
| K91 | Cerebrovascular dis. | D98 | Cholecystitis/cholelithiasis | 1.247 |
| K92 | Atherosclerosis/peripheral vascular dis | D98 | Cholecystitis/cholelithiasis | 1.261 |
| N93 | Carpal tunnel syndrome | D98 | Cholecystitis/cholelithiasis | 1.26 |
| N94 | Peripheral neuritis/neuropathy | D98 | Cholecystitis/cholelithiasis | 1.353 |
| T83/82 | Obesity/overweight | D98 | Cholecystitis/cholelithiasis | 1.55 |
| T90 | Type 2 Diabetes mellitus | D98 | Cholecystitis/cholelithiasis | 1.341 |
| T99 | Endocrine/metabolic/nutrition dis. other | D98 | Cholecystitis/cholelithiasis | 1.296 |
| U88 | Glomerulonephritis/nephrosis | D98 | Cholecystitis/cholelithiasis | 1.301 |
| U99.01 | Chronic renal failure | D98 | Cholecystitis/cholelithiasis | 1.353 |
| X77 | Female genital ca, other | D98 | Cholecystitis/cholelithiasis | 1.285 |
| B81 | Anaemia vitamin B12/folate deficiency | D99 | Dis. digestive system, other | 1.929 |
| B82 | Anaemia other, unspecified | D99 | Dis. digestive system, other | 2.063 |
| D76 | Pancreas Ca. | D99 | Dis. digestive system, other | 5.72 |
| D77 | Digestive ca. other/NOS | D99 | Dis. digestive system, other | 1.879 |
| D97 | Liver dis. NOS | D99 | Dis. digestive system, other | 2.209 |
| D98 | Cholecystitis/cholelithiasis | D99 | Dis. digestive system, other | 2.73 |
| F83 | Retinopathy | D99 | Dis. digestive system, other | 1.7 |
| F84 | Macular degeneration | D99 | Dis. digestive system, other | 1.376 |
| K74 | Ischaemic heart dis. with angina | D99 | Dis. digestive system, other | 1.631 |
| K75 | Acute myocardial infarction | D99 | Dis. digestive system, other | 1.428 |
| K76 | Ischaemic heart dis. without angina | D99 | Dis. digestive system, other | 1.534 |
| K77 | Heart failure | D99 | Dis. digestive system, other | 1.615 |
| K78 | Atrial Fibrillation/flutter | D99 | Dis. digestive system, other | 1.41 |
| K82 | Pulmonary heart dis. | D99 | Dis. digestive system, other | 1.815 |
| K84 | Heart dis. other | D99 | Dis. digestive system, other | 1.251 |
| K87 | Hypertension complicated | D99 | Dis. digestive system, other | 1.433 |
| K89 | Transient cerebral ischaemia | D99 | Dis. digestive system, other | 1.35 |
| K90 | Stroke | D99 | Dis. digestive system, other | 1.432 |
| K91 | Cerebrovascular dis. | D99 | Dis. digestive system, other | 1.667 |
| K92 | Atherosclerosis/peripheral vascular dis | D99 | Dis. digestive system, other | 2.1 |
| N94 | Peripheral neuritis/neuropathy | D99 | Dis. digestive system, other | 1.976 |
| P71 | Psychosis organic, other | D99 | Dis. digestive system, other | 1.359 |
| T89 | Type 1 Diabetes mellitus | D99 | Dis. digestive system, other | 2.939 |
| T90 | Type 2 Diabetes mellitus | D99 | Dis. digestive system, other | 1.587 |
| T99 | Endocrine/metabolic/nutrition dis. other | D99 | Dis. digestive system, other | 1.53 |
| U88 | Glomerulonephritis/nephrosis | D99 | Dis. digestive system, other | 1.61 |
| U99.01 | Chronic renal failure | D99 | Dis. digestive system, other | 1.62 |
| B82 | Anaemia other, unspecified | F83 | Retinopathy | 2.34 |
| D97 | Liver dis. NOS | F83 | Retinopathy | 1.307 |
| D98 | Cholecystitis/cholelithiasis | F83 | Retinopathy | 1.33 |
| F84 | Macular degeneration | F83 | Retinopathy | 2.32 |
| F92 | Cataract | F83 | Retinopathy | 1.997 |
| K74 | Ischaemic heart dis. with angina | F83 | Retinopathy | 1.996 |
| K76 | Ischaemic heart dis. without angina | F83 | Retinopathy | 2.459 |
| K86 | Hypertension uncomplicated | F83 | Retinopathy | 3.352 |
| K90 | Stroke | F83 | Retinopathy | 2.031 |
| K92 | Atherosclerosis/peripheral vascular dis | F83 | Retinopathy | 3.212 |
| N93 | Carpal tunnel syndrome | F83 | Retinopathy | 1.53 |
| N94 | Peripheral neuritis/neuropathy | F83 | Retinopathy | 5.704 |
| T83/82 | Obesity/overweight | F83 | Retinopathy | 2.003 |
| T89 | Type 1 Diabetes mellitus | F83 | Retinopathy | 25.336 |
| T90 | Type 2 Diabetes mellitus | F83 | Retinopathy | 23.829 |
| T93 | Lipid disorder | F83 | Retinopathy | 1.476 |
| U88 | Glomerulonephritis/nephrosis | F83 | Retinopathy | 5.951 |
| X77 | Female genital ca, other | F83 | Retinopathy | 1.26 |
| B82 | Anaemia other, unspecified | F84 | Macular degeneration | 1.223 |
| D97 | Liver dis. NOS | F84 | Macular degeneration | 1.267 |
| D98 | Cholecystitis/cholelithiasis | F84 | Macular degeneration | 1.303 |
| D99 | Dis. digestive system, other | F84 | Macular degeneration | 1.376 |
| F92 | Cataract | F84 | Macular degeneration | 2.199 |
| K74 | Ischaemic heart dis. with angina | F84 | Macular degeneration | 1.289 |
| K76 | Ischaemic heart dis. without angina | F84 | Macular degeneration | 1.203 |
| K84 | Heart dis. other | F84 | Macular degeneration | 1.421 |
| K86 | Hypertension uncomplicated | F84 | Macular degeneration | 1.264 |
| K87 | Hypertension complicated | F84 | Macular degeneration | 1.387 |
| K89 | Transient cerebral ischaemia | F84 | Macular degeneration | 1.352 |
| K91 | Cerebrovascular dis. | F84 | Macular degeneration | 1.406 |
| K92 | Atherosclerosis/peripheral vascular dis | F84 | Macular degeneration | 1.354 |
| N94 | Peripheral neuritis/neuropathy | F84 | Macular degeneration | 1.479 |
| T90 | Type 2 Diabetes mellitus | F84 | Macular degeneration | 1.26 |
| T99 | Endocrine/metabolic/nutrition dis. other | F84 | Macular degeneration | 1.205 |
| U99.01 | Chronic renal failure | F84 | Macular degeneration | 1.34 |
| B81 | Anaemia vitamin B12/folate deficiency | F92 | Cataract | 1.277 |
| B82 | Anaemia other, unspecified | F92 | Cataract | 1.398 |
| D98 | Cholecystitis/cholelithiasis | F92 | Cataract | 1.39 |
| D99 | Dis. digestive system, other | F92 | Cataract | 1.383 |
| F83 | Retinopathy | F92 | Cataract | 1.997 |
| F93 | Glaucoma | F92 | Cataract | 1.899 |
| K74 | Ischaemic heart dis. with angina | F92 | Cataract | 1.37 |
| K76 | Ischaemic heart dis. without angina | F92 | Cataract | 1.23 |
| K78 | Atrial Fibrillation/flutter | F92 | Cataract | 1.239 |
| K86 | Hypertension uncomplicated | F92 | Cataract | 1.353 |
| K87 | Hypertension complicated | F92 | Cataract | 1.415 |
| K89 | Transient cerebral ischaemia | F92 | Cataract | 1.323 |
| K91 | Cerebrovascular dis. | F92 | Cataract | 1.323 |
| N93 | Carpal tunnel syndrome | F92 | Cataract | 1.384 |
| N94 | Peripheral neuritis/neuropathy | F92 | Cataract | 1.565 |
| T83/82 | Obesity/overweight | F92 | Cataract | 1.277 |
| T89 | Type 1 Diabetes mellitus | F92 | Cataract | 1.467 |
| T90 | Type 2 Diabetes mellitus | F92 | Cataract | 1.379 |
| T92 | Gout | F92 | Cataract | 1.297 |
| U88 | Glomerulonephritis/nephrosis | F92 | Cataract | 1.457 |
| F83 | Retinopathy | F93 | Glaucoma | 1.829 |
| F84 | Macular degeneration | F93 | Glaucoma | 1.687 |
| K84 | Heart dis. other | F93 | Glaucoma | 1.214 |
| K86 | Hypertension uncomplicated | F93 | Glaucoma | 1.427 |
| K92 | Atherosclerosis/peripheral vascular dis | F93 | Glaucoma | 1.254 |
| N93 | Carpal tunnel syndrome | F93 | Glaucoma | 1.224 |
| N94 | Peripheral neuritis/neuropathy | F93 | Glaucoma | 1.391 |
| T89 | Type 1 Diabetes mellitus | F93 | Glaucoma | 1.912 |
| T90 | Type 2 Diabetes mellitus | F93 | Glaucoma | 1.506 |
| T93 | Lipid disorder | F93 | Glaucoma | 1.227 |
| U88 | Glomerulonephritis/nephrosis | F93 | Glaucoma | 1.334 |
| D98 | Cholecystitis/cholelithiasis | K74 | Ischaemic heart dis. with angina | 1.413 |
| F83 | Retinopathy | K74 | Ischaemic heart dis. with angina | 1.996 |
| F92 | Cataract | K74 | Ischaemic heart dis. with angina | 1.37 |
| K75 | Acute myocardial infarction | K74 | Ischaemic heart dis. with angina | 5.429 |
| K78 | Atrial Fibrillation/flutter | K74 | Ischaemic heart dis. with angina | 1.957 |
| K84 | Heart dis. other | K74 | Ischaemic heart dis. with angina | 1.654 |
| K86 | Hypertension uncomplicated | K74 | Ischaemic heart dis. with angina | 1.9 |
| K87 | Hypertension complicated | K74 | Ischaemic heart dis. with angina | 1.871 |
| K89 | Transient cerebral ischaemia | K74 | Ischaemic heart dis. with angina | 1.428 |
| K90 | Stroke | K74 | Ischaemic heart dis. with angina | 1.357 |
| K91 | Cerebrovascular dis. | K74 | Ischaemic heart dis. with angina | 1.568 |
| K92 | Atherosclerosis/peripheral vascular dis | K74 | Ischaemic heart dis. with angina | 2.059 |
| N93 | Carpal tunnel syndrome | K74 | Ischaemic heart dis. with angina | 1.29 |
| T83/82 | Obesity/overweight | K74 | Ischaemic heart dis. with angina | 1.458 |
| T89 | Type 1 Diabetes mellitus | K74 | Ischaemic heart dis. with angina | 1.747 |
| T93 | Lipid disorder | K74 | Ischaemic heart dis. with angina | 1.789 |
| T99 | Endocrine/metabolic/nutrition dis. other | K74 | Ischaemic heart dis. with angina | 1.279 |
| B82 | Anaemia other, unspecified | K75 | Acute myocardial infarction | 1.615 |
| D98 | Cholecystitis/cholelithiasis | K75 | Acute myocardial infarction | 1.25 |
| F83 | Retinopathy | K75 | Acute myocardial infarction | 2.183 |
| K77 | Heart failure | K75 | Acute myocardial infarction | 3.272 |
| K84 | Heart dis. other | K75 | Acute myocardial infarction | 1.403 |
| K86 | Hypertension uncomplicated | K75 | Acute myocardial infarction | 1.506 |
| K89 | Transient cerebral ischaemia | K75 | Acute myocardial infarction | 1.318 |
| K90 | Stroke | K75 | Acute myocardial infarction | 1.574 |
| K92 | Atherosclerosis/peripheral vascular dis | K75 | Acute myocardial infarction | 2.353 |
| N94 | Peripheral neuritis/neuropathy | K75 | Acute myocardial infarction | 1.479 |
| T83/82 | Obesity/overweight | K75 | Acute myocardial infarction | 1.264 |
| T89 | Type 1 Diabetes mellitus | K75 | Acute myocardial infarction | 2.393 |
| T90 | Type 2 Diabetes mellitus | K75 | Acute myocardial infarction | 2.066 |
| T93 | Lipid disorder | K75 | Acute myocardial infarction | 1.728 |
| U88 | Glomerulonephritis/nephrosis | K75 | Acute myocardial infarction | 1.717 |
| U99.01 | Chronic renal failure | K75 | Acute myocardial infarction | 1.932 |
| B82 | Anaemia other, unspecified | K76 | Ischaemic heart dis. without angina | 1.748 |
| D98 | Cholecystitis/cholelithiasis | K76 | Ischaemic heart dis. without angina | 1.313 |
| F83 | Retinopathy | K76 | Ischaemic heart dis. without angina | 2.459 |
| K74 | Ischaemic heart dis. with angina | K76 | Ischaemic heart dis. without angina | 7.594 |
| K75 | Acute myocardial infarction | K76 | Ischaemic heart dis. without angina | 11.301 |
| K84 | Heart dis. other | K76 | Ischaemic heart dis. without angina | 1.671 |
| K86 | Hypertension uncomplicated | K76 | Ischaemic heart dis. without angina | 1.782 |
| K87 | Hypertension complicated | K76 | Ischaemic heart dis. without angina | 1.74 |
| K89 | Transient cerebral ischaemia | K76 | Ischaemic heart dis. without angina | 1.399 |
| K90 | Stroke | K76 | Ischaemic heart dis. without angina | 1.564 |
| K91 | Cerebrovascular dis. | K76 | Ischaemic heart dis. without angina | 1.847 |
| N94 | Peripheral neuritis/neuropathy | K76 | Ischaemic heart dis. without angina | 1.683 |
| T83/82 | Obesity/overweight | K76 | Ischaemic heart dis. without angina | 1.36 |
| T89 | Type 1 Diabetes mellitus | K76 | Ischaemic heart dis. without angina | 2.439 |
| T90 | Type 2 Diabetes mellitus | K76 | Ischaemic heart dis. without angina | 2.161 |
| T93 | Lipid disorder | K76 | Ischaemic heart dis. without angina | 1.808 |
| T99 | Endocrine/metabolic/nutrition dis. other | K76 | Ischaemic heart dis. without angina | 1.302 |
| U99.01 | Chronic renal failure | K76 | Ischaemic heart dis. without angina | 1.964 |
| B81 | Anaemia vitamin B12/folate deficiency | K77 | Heart failure | 1.381 |
| D99 | Dis. digestive system, other | K77 | Heart failure | 1.615 |
| F83 | Retinopathy | K77 | Heart failure | 2.173 |
| K74 | Ischaemic heart dis. with angina | K77 | Heart failure | 2.554 |
| K75 | Acute myocardial infarction | K77 | Heart failure | 3.272 |
| K76 | Ischaemic heart dis. without angina | K77 | Heart failure | 3.42 |
| K78 | Atrial Fibrillation/flutter | K77 | Heart failure | 6.206 |
| K84 | Heart dis. other | K77 | Heart failure | 2.404 |
| K86 | Hypertension uncomplicated | K77 | Heart failure | 1.774 |
| K87 | Hypertension complicated | K77 | Heart failure | 2.58 |
| K89 | Transient cerebral ischaemia | K77 | Heart failure | 1.357 |
| K91 | Cerebrovascular dis. | K77 | Heart failure | 1.36 |
| K92 | Atherosclerosis/peripheral vascular dis | K77 | Heart failure | 1.924 |
| N94 | Peripheral neuritis/neuropathy | K77 | Heart failure | 1.644 |
| T83/82 | Obesity/overweight | K77 | Heart failure | 1.677 |
| T89 | Type 1 Diabetes mellitus | K77 | Heart failure | 2.117 |
| T90 | Type 2 Diabetes mellitus | K77 | Heart failure | 1.867 |
| T92 | Gout | K77 | Heart failure | 1.922 |
| T99 | Endocrine/metabolic/nutrition dis. other | K77 | Heart failure | 1.773 |
| U88 | Glomerulonephritis/nephrosis | K77 | Heart failure | 1.727 |
| U99.01 | Chronic renal failure | K77 | Heart failure | 2.561 |
| B82 | Anaemia other, unspecified | K78 | Atrial Fibrillation/flutter | 1.638 |
| D99 | Dis. digestive system, other | K78 | Atrial Fibrillation/flutter | 1.41 |
| F83 | Retinopathy | K78 | Atrial Fibrillation/flutter | 1.35 |
| F92 | Cataract | K78 | Atrial Fibrillation/flutter | 1.239 |
| K74 | Ischaemic heart dis. with angina | K78 | Atrial Fibrillation/flutter | 1.957 |
| K75 | Acute myocardial infarction | K78 | Atrial Fibrillation/flutter | 1.72 |
| K76 | Ischaemic heart dis. without angina | K78 | Atrial Fibrillation/flutter | 2.024 |
| K77 | Heart failure | K78 | Atrial Fibrillation/flutter | 6.206 |
| K84 | Heart dis. other | K78 | Atrial Fibrillation/flutter | 1.748 |
| K86 | Hypertension uncomplicated | K78 | Atrial Fibrillation/flutter | 1.737 |
| K87 | Hypertension complicated | K78 | Atrial Fibrillation/flutter | 2.397 |
| K89 | Transient cerebral ischaemia | K78 | Atrial Fibrillation/flutter | 1.619 |
| K91 | Cerebrovascular dis. | K78 | Atrial Fibrillation/flutter | 1.37 |
| K92 | Atherosclerosis/peripheral vascular dis | K78 | Atrial Fibrillation/flutter | 1.583 |
| P71 | Psychosis organic, other | K78 | Atrial Fibrillation/flutter | 1.292 |
| T83/82 | Obesity/overweight | K78 | Atrial Fibrillation/flutter | 1.54 |
| T99 | Endocrine/metabolic/nutrition dis. other | K78 | Atrial Fibrillation/flutter | 1.541 |
| U99.01 | Chronic renal failure | K78 | Atrial Fibrillation/flutter | 1.881 |
| B78 | Hereditary haemolytic anaemia | K82 | Pulmonary heart dis. | 1.609 |
| B81 | Anaemia vitamin B12/folate deficiency | K82 | Pulmonary heart dis. | 1.424 |
| B82 | Anaemia other, unspecified | K82 | Pulmonary heart dis. | 2.316 |
| D97 | Liver dis. NOS | K82 | Pulmonary heart dis. | 1.801 |
| D98 | Cholecystitis/cholelithiasis | K82 | Pulmonary heart dis. | 1.568 |
| D99 | Dis. digestive system, other | K82 | Pulmonary heart dis. | 1.815 |
| F83 | Retinopathy | K82 | Pulmonary heart dis. | 1.79 |
| F92 | Cataract | K82 | Pulmonary heart dis. | 1.407 |
| K74 | Ischaemic heart dis. with angina | K82 | Pulmonary heart dis. | 1.856 |
| K75 | Acute myocardial infarction | K82 | Pulmonary heart dis. | 1.696 |
| K76 | Ischaemic heart dis. without angina | K82 | Pulmonary heart dis. | 2.169 |
| K77 | Heart failure | K82 | Pulmonary heart dis. | 9.551 |
| K78 | Atrial Fibrillation/flutter | K82 | Pulmonary heart dis. | 8.088 |
| K84 | Heart dis. other | K82 | Pulmonary heart dis. | 2.583 |
| K86 | Hypertension uncomplicated | K82 | Pulmonary heart dis. | 1.608 |
| K87 | Hypertension complicated | K82 | Pulmonary heart dis. | 4.234 |
| K90 | Stroke | K82 | Pulmonary heart dis. | 1.227 |
| K92 | Atherosclerosis/peripheral vascular dis | K82 | Pulmonary heart dis. | 1.893 |
| N94 | Peripheral neuritis/neuropathy | K82 | Pulmonary heart dis. | 1.531 |
| T83/82 | Obesity/overweight | K82 | Pulmonary heart dis. | 1.81 |
| T90 | Type 2 Diabetes mellitus | K82 | Pulmonary heart dis. | 1.647 |
| T92 | Gout | K82 | Pulmonary heart dis. | 2.056 |
| U88 | Glomerulonephritis/nephrosis | K82 | Pulmonary heart dis. | 1.833 |
| U99.01 | Chronic renal failure | K82 | Pulmonary heart dis. | 2.569 |
| B82 | Anaemia other, unspecified | K84 | Heart dis. other | 1.466 |
| D98 | Cholecystitis/cholelithiasis | K84 | Heart dis. other | 1.305 |
| F83 | Retinopathy | K84 | Heart dis. other | 1.901 |
| F92 | Cataract | K84 | Heart dis. other | 1.483 |
| F93 | Glaucoma | K84 | Heart dis. other | 1.214 |
| K74 | Ischaemic heart dis. with angina | K84 | Heart dis. other | 1.654 |
| K86 | Hypertension uncomplicated | K84 | Heart dis. other | 2.14 |
| K87 | Hypertension complicated | K84 | Heart dis. other | 2.8 |
| K89 | Transient cerebral ischaemia | K84 | Heart dis. other | 1.264 |
| K91 | Cerebrovascular dis. | K84 | Heart dis. other | 1.285 |
| K92 | Atherosclerosis/peripheral vascular dis | K84 | Heart dis. other | 1.384 |
| N94 | Peripheral neuritis/neuropathy | K84 | Heart dis. other | 1.372 |
| P71 | Psychosis organic, other | K84 | Heart dis. other | 1.239 |
| T83/82 | Obesity/overweight | K84 | Heart dis. other | 1.51 |
| T90 | Type 2 Diabetes mellitus | K84 | Heart dis. other | 1.613 |
| T92 | Gout | K84 | Heart dis. other | 1.45 |
| T93 | Lipid disorder | K84 | Heart dis. other | 1.253 |
| T99 | Endocrine/metabolic/nutrition dis. other | K84 | Heart dis. other | 1.49 |
| U99.01 | Chronic renal failure | K84 | Heart dis. other | 1.784 |
| B82 | Anaemia other, unspecified | K86 | Hypertension uncomplicated | 1.421 |
| D98 | Cholecystitis/cholelithiasis | K86 | Hypertension uncomplicated | 1.247 |
| F84 | Macular degeneration | K86 | Hypertension uncomplicated | 1.264 |
| F93 | Glaucoma | K86 | Hypertension uncomplicated | 1.427 |
| K89 | Transient cerebral ischaemia | K86 | Hypertension uncomplicated | 1.687 |
| K90 | Stroke | K86 | Hypertension uncomplicated | 1.937 |
| K91 | Cerebrovascular dis. | K86 | Hypertension uncomplicated | 1.891 |
| K92 | Atherosclerosis/peripheral vascular dis | K86 | Hypertension uncomplicated | 1.714 |
| T83/82 | Obesity/overweight | K86 | Hypertension uncomplicated | 2.857 |
| T89 | Type 1 Diabetes mellitus | K86 | Hypertension uncomplicated | 1.55 |
| T90 | Type 2 Diabetes mellitus | K86 | Hypertension uncomplicated | 2.759 |
| T93 | Lipid disorder | K86 | Hypertension uncomplicated | 1.686 |
| T99 | Endocrine/metabolic/nutrition dis. other | K86 | Hypertension uncomplicated | 1.985 |
| U88 | Glomerulonephritis/nephrosis | K86 | Hypertension uncomplicated | 3.021 |
| B81 | Anaemia vitamin B12/folate deficiency | K87 | Hypertension complicated | 1.258 |
| B82 | Anaemia other, unspecified | K87 | Hypertension complicated | 1.659 |
| D97 | Liver dis. NOS | K87 | Hypertension complicated | 1.545 |
| D98 | Cholecystitis/cholelithiasis | K87 | Hypertension complicated | 1.352 |
| D99 | Dis. digestive system, other | K87 | Hypertension complicated | 1.433 |
| F83 | Retinopathy | K87 | Hypertension complicated | 2.188 |
| F84 | Macular degeneration | K87 | Hypertension complicated | 1.387 |
| F92 | Cataract | K87 | Hypertension complicated | 1.415 |
| F93 | Glaucoma | K87 | Hypertension complicated | 1.239 |
| K74 | Ischaemic heart dis. with angina | K87 | Hypertension complicated | 1.871 |
| K75 | Acute myocardial infarction | K87 | Hypertension complicated | 1.206 |
| K76 | Ischaemic heart dis. without angina | K87 | Hypertension complicated | 1.74 |
| K86 | Hypertension uncomplicated | K87 | Hypertension complicated | 9.578 |
| K89 | Transient cerebral ischaemia | K87 | Hypertension complicated | 1.465 |
| K90 | Stroke | K87 | Hypertension complicated | 1.506 |
| K91 | Cerebrovascular dis. | K87 | Hypertension complicated | 1.815 |
| K92 | Atherosclerosis/peripheral vascular dis | K87 | Hypertension complicated | 1.657 |
| N94 | Peripheral neuritis/neuropathy | K87 | Hypertension complicated | 1.473 |
| T83/82 | Obesity/overweight | K87 | Hypertension complicated | 2.021 |
| T89 | Type 1 Diabetes mellitus | K87 | Hypertension complicated | 1.282 |
| T90 | Type 2 Diabetes mellitus | K87 | Hypertension complicated | 1.692 |
| T92 | Gout | K87 | Hypertension complicated | 1.818 |
| T93 | Lipid disorder | K87 | Hypertension complicated | 1.444 |
| U88 | Glomerulonephritis/nephrosis | K87 | Hypertension complicated | 2.139 |
| U99.01 | Chronic renal failure | K87 | Hypertension complicated | 2.332 |
| B81 | Anaemia vitamin B12/folate deficiency | K89 | Transient cerebral ischaemia | 1.299 |
| B82 | Anaemia other, unspecified | K89 | Transient cerebral ischaemia | 1.358 |
| D98 | Cholecystitis/cholelithiasis | K89 | Transient cerebral ischaemia | 1.23 |
| F83 | Retinopathy | K89 | Transient cerebral ischaemia | 1.567 |
| F84 | Macular degeneration | K89 | Transient cerebral ischaemia | 1.352 |
| F92 | Cataract | K89 | Transient cerebral ischaemia | 1.323 |
| K74 | Ischaemic heart dis. with angina | K89 | Transient cerebral ischaemia | 1.428 |
| K75 | Acute myocardial infarction | K89 | Transient cerebral ischaemia | 1.318 |
| K76 | Ischaemic heart dis. without angina | K89 | Transient cerebral ischaemia | 1.399 |
| K84 | Heart dis. other | K89 | Transient cerebral ischaemia | 1.264 |
| K86 | Hypertension uncomplicated | K89 | Transient cerebral ischaemia | 1.687 |
| K87 | Hypertension complicated | K89 | Transient cerebral ischaemia | 1.465 |
| K90 | Stroke | K89 | Transient cerebral ischaemia | 2.399 |
| K92 | Atherosclerosis/peripheral vascular dis | K89 | Transient cerebral ischaemia | 1.739 |
| N94 | Peripheral neuritis/neuropathy | K89 | Transient cerebral ischaemia | 1.438 |
| T89 | Type 1 Diabetes mellitus | K89 | Transient cerebral ischaemia | 1.265 |
| T92 | Gout | K89 | Transient cerebral ischaemia | 1.243 |
| T93 | Lipid disorder | K89 | Transient cerebral ischaemia | 1.311 |
| U99.01 | Chronic renal failure | K89 | Transient cerebral ischaemia | 1.402 |
| B81 | Anaemia vitamin B12/folate deficiency | K90 | Stroke | 1.438 |
| B82 | Anaemia other, unspecified | K90 | Stroke | 1.446 |
| D99 | Dis. digestive system, other | K90 | Stroke | 1.432 |
| F83 | Retinopathy | K90 | Stroke | 2.031 |
| K74 | Ischaemic heart dis. with angina | K90 | Stroke | 1.357 |
| K75 | Acute myocardial infarction | K90 | Stroke | 1.574 |
| K77 | Heart failure | K90 | Stroke | 1.594 |
| K78 | Atrial Fibrillation/flutter | K90 | Stroke | 2.177 |
| K86 | Hypertension uncomplicated | K90 | Stroke | 1.937 |
| K89 | Transient cerebral ischaemia | K90 | Stroke | 2.399 |
| K92 | Atherosclerosis/peripheral vascular dis | K90 | Stroke | 1.951 |
| P71 | Psychosis organic, other | K90 | Stroke | 1.71 |
| T89 | Type 1 Diabetes mellitus | K90 | Stroke | 1.922 |
| T90 | Type 2 Diabetes mellitus | K90 | Stroke | 1.644 |
| U88 | Glomerulonephritis/nephrosis | K90 | Stroke | 1.484 |
| B81 | Anaemia vitamin B12/folate deficiency | K91 | Cerebrovascular dis. | 1.379 |
| B82 | Anaemia other, unspecified | K91 | Cerebrovascular dis. | 1.565 |
| D98 | Cholecystitis/cholelithiasis | K91 | Cerebrovascular dis. | 1.247 |
| D99 | Dis. digestive system, other | K91 | Cerebrovascular dis. | 1.667 |
| F83 | Retinopathy | K91 | Cerebrovascular dis. | 1.879 |
| F84 | Macular degeneration | K91 | Cerebrovascular dis. | 1.406 |
| F92 | Cataract | K91 | Cerebrovascular dis. | 1.323 |
| F93 | Glaucoma | K91 | Cerebrovascular dis. | 1.251 |
| K74 | Ischaemic heart dis. with angina | K91 | Cerebrovascular dis. | 1.568 |
| K75 | Acute myocardial infarction | K91 | Cerebrovascular dis. | 1.519 |
| K76 | Ischaemic heart dis. without angina | K91 | Cerebrovascular dis. | 1.847 |
| K78 | Atrial Fibrillation/flutter | K91 | Cerebrovascular dis. | 1.37 |
| K84 | Heart dis. other | K91 | Cerebrovascular dis. | 1.285 |
| K86 | Hypertension uncomplicated | K91 | Cerebrovascular dis. | 1.891 |
| K87 | Hypertension complicated | K91 | Cerebrovascular dis. | 1.815 |
| K89 | Transient cerebral ischaemia | K91 | Cerebrovascular dis. | 3.717 |
| K90 | Stroke | K91 | Cerebrovascular dis. | 5.723 |
| K92 | Atherosclerosis/peripheral vascular dis | K91 | Cerebrovascular dis. | 3.098 |
| P71 | Psychosis organic, other | K91 | Cerebrovascular dis. | 1.949 |
| T89 | Type 1 Diabetes mellitus | K91 | Cerebrovascular dis. | 1.604 |
| T90 | Type 2 Diabetes mellitus | K91 | Cerebrovascular dis. | 1.416 |
| T93 | Lipid disorder | K91 | Cerebrovascular dis. | 1.459 |
| T99 | Endocrine/metabolic/nutrition dis. other | K91 | Cerebrovascular dis. | 1.201 |
| U88 | Glomerulonephritis/nephrosis | K91 | Cerebrovascular dis. | 1.625 |
| U99.01 | Chronic renal failure | K91 | Cerebrovascular dis. | 1.55 |
| B82 | Anaemia other, unspecified | K92 | Atherosclerosis/peripheral vascular dis | 1.857 |
| D77 | Digestive ca. other/NOS | K92 | Atherosclerosis/peripheral vascular dis | 1.235 |
| D97 | Liver dis. NOS | K92 | Atherosclerosis/peripheral vascular dis | 1.237 |
| D98 | Cholecystitis/cholelithiasis | K92 | Atherosclerosis/peripheral vascular dis | 1.261 |
| F83 | Retinopathy | K92 | Atherosclerosis/peripheral vascular dis | 3.212 |
| F84 | Macular degeneration | K92 | Atherosclerosis/peripheral vascular dis | 1.354 |
| F92 | Cataract | K92 | Atherosclerosis/peripheral vascular dis | 1.408 |
| F93 | Glaucoma | K92 | Atherosclerosis/peripheral vascular dis | 1.254 |
| K74 | Ischaemic heart dis. with angina | K92 | Atherosclerosis/peripheral vascular dis | 2.059 |
| K75 | Acute myocardial infarction | K92 | Atherosclerosis/peripheral vascular dis | 2.353 |
| K76 | Ischaemic heart dis. without angina | K92 | Atherosclerosis/peripheral vascular dis | 2.464 |
| K77 | Heart failure | K92 | Atherosclerosis/peripheral vascular dis | 1.924 |
| K82 | Pulmonary heart dis. | K92 | Atherosclerosis/peripheral vascular dis | 1.893 |
| K84 | Heart dis. other | K92 | Atherosclerosis/peripheral vascular dis | 1.384 |
| K86 | Hypertension uncomplicated | K92 | Atherosclerosis/peripheral vascular dis | 1.714 |
| K89 | Transient cerebral ischaemia | K92 | Atherosclerosis/peripheral vascular dis | 1.739 |
| K90 | Stroke | K92 | Atherosclerosis/peripheral vascular dis | 1.951 |
| K91 | Cerebrovascular dis. | K92 | Atherosclerosis/peripheral vascular dis | 3.098 |
| N93 | Carpal tunnel syndrome | K92 | Atherosclerosis/peripheral vascular dis | 1.259 |
| N94 | Peripheral neuritis/neuropathy | K92 | Atherosclerosis/peripheral vascular dis | 2.752 |
| P71 | Psychosis organic, other | K92 | Atherosclerosis/peripheral vascular dis | 1.294 |
| T89 | Type 1 Diabetes mellitus | K92 | Atherosclerosis/peripheral vascular dis | 3.053 |
| T90 | Type 2 Diabetes mellitus | K92 | Atherosclerosis/peripheral vascular dis | 2.039 |
| T93 | Lipid disorder | K92 | Atherosclerosis/peripheral vascular dis | 1.42 |
| T99 | Endocrine/metabolic/nutrition dis. other | K92 | Atherosclerosis/peripheral vascular dis | 1.318 |
| U88 | Glomerulonephritis/nephrosis | K92 | Atherosclerosis/peripheral vascular dis | 2.188 |
| U99.01 | Chronic renal failure | K92 | Atherosclerosis/peripheral vascular dis | 2.094 |
| D97 | Liver dis. NOS | N93 | Carpal tunnel syndrome | 1.38 |
| D98 | Cholecystitis/cholelithiasis | N93 | Carpal tunnel syndrome | 1.26 |
| F83 | Retinopathy | N93 | Carpal tunnel syndrome | 1.53 |
| F84 | Macular degeneration | N93 | Carpal tunnel syndrome | 1.29 |
| F92 | Cataract | N93 | Carpal tunnel syndrome | 1.384 |
| F93 | Glaucoma | N93 | Carpal tunnel syndrome | 1.224 |
| K87 | Hypertension complicated | N93 | Carpal tunnel syndrome | 1.246 |
| K92 | Atherosclerosis/peripheral vascular dis | N93 | Carpal tunnel syndrome | 1.259 |
| N94 | Peripheral neuritis/neuropathy | N93 | Carpal tunnel syndrome | 2.549 |
| T83/82 | Obesity/overweight | N93 | Carpal tunnel syndrome | 1.588 |
| T89 | Type 1 Diabetes mellitus | N93 | Carpal tunnel syndrome | 1.464 |
| T90 | Type 2 Diabetes mellitus | N93 | Carpal tunnel syndrome | 1.24 |
| T93 | Lipid disorder | N93 | Carpal tunnel syndrome | 1.256 |
| B81 | Anaemia vitamin B12/folate deficiency | N94 | Peripheral neuritis/neuropathy | 1.887 |
| B82 | Anaemia other, unspecified | N94 | Peripheral neuritis/neuropathy | 1.682 |
| D77 | Digestive ca. other/NOS | N94 | Peripheral neuritis/neuropathy | 1.291 |
| D97 | Liver dis. NOS | N94 | Peripheral neuritis/neuropathy | 1.708 |
| D98 | Cholecystitis/cholelithiasis | N94 | Peripheral neuritis/neuropathy | 1.353 |
| D99 | Dis. digestive system, other | N94 | Peripheral neuritis/neuropathy | 1.976 |
| F83 | Retinopathy | N94 | Peripheral neuritis/neuropathy | 5.704 |
| F84 | Macular degeneration | N94 | Peripheral neuritis/neuropathy | 1.479 |
| F93 | Glaucoma | N94 | Peripheral neuritis/neuropathy | 1.391 |
| K74 | Ischaemic heart dis. with angina | N94 | Peripheral neuritis/neuropathy | 1.639 |
| K77 | Heart failure | N94 | Peripheral neuritis/neuropathy | 1.644 |
| K84 | Heart dis. other | N94 | Peripheral neuritis/neuropathy | 1.372 |
| K86 | Hypertension uncomplicated | N94 | Peripheral neuritis/neuropathy | 1.301 |
| K87 | Hypertension complicated | N94 | Peripheral neuritis/neuropathy | 1.473 |
| K89 | Transient cerebral ischaemia | N94 | Peripheral neuritis/neuropathy | 1.438 |
| K90 | Stroke | N94 | Peripheral neuritis/neuropathy | 1.539 |
| K91 | Cerebrovascular dis. | N94 | Peripheral neuritis/neuropathy | 1.737 |
| K92 | Atherosclerosis/peripheral vascular dis | N94 | Peripheral neuritis/neuropathy | 2.752 |
| N93 | Carpal tunnel syndrome | N94 | Peripheral neuritis/neuropathy | 2.549 |
| P71 | Psychosis organic, other | N94 | Peripheral neuritis/neuropathy | 1.388 |
| T83/82 | Obesity/overweight | N94 | Peripheral neuritis/neuropathy | 1.39 |
| T89 | Type 1 Diabetes mellitus | N94 | Peripheral neuritis/neuropathy | 5.224 |
| T90 | Type 2 Diabetes mellitus | N94 | Peripheral neuritis/neuropathy | 2.704 |
| T99 | Endocrine/metabolic/nutrition dis. other | N94 | Peripheral neuritis/neuropathy | 1.206 |
| U88 | Glomerulonephritis/nephrosis | N94 | Peripheral neuritis/neuropathy | 2.713 |
| X77 | Female genital ca, other | N94 | Peripheral neuritis/neuropathy | 1.224 |
| B81 | Anaemia vitamin B12/folate deficiency | P71 | Psychosis organic, other | 1.671 |
| D99 | Dis. digestive system, other | P71 | Psychosis organic, other | 1.359 |
| F83 | Retinopathy | P71 | Psychosis organic, other | 1.436 |
| F84 | Macular degeneration | P71 | Psychosis organic, other | 1.429 |
| F92 | Cataract | P71 | Psychosis organic, other | 1.41 |
| K76 | Ischaemic heart dis. without angina | P71 | Psychosis organic, other | 1.217 |
| K77 | Heart failure | P71 | Psychosis organic, other | 1.225 |
| K78 | Atrial Fibrillation/flutter | P71 | Psychosis organic, other | 1.292 |
| K87 | Hypertension complicated | P71 | Psychosis organic, other | 1.315 |
| K89 | Transient cerebral ischaemia | P71 | Psychosis organic, other | 1.56 |
| K90 | Stroke | P71 | Psychosis organic, other | 1.71 |
| K91 | Cerebrovascular dis. | P71 | Psychosis organic, other | 1.949 |
| N94 | Peripheral neuritis/neuropathy | P71 | Psychosis organic, other | 1.388 |
| U88 | Glomerulonephritis/nephrosis | P71 | Psychosis organic, other | 1.28 |
| U99.01 | Chronic renal failure | P71 | Psychosis organic, other | 1.399 |
| P71 | Psychosis organic, other | P72 | Schizophrenia | 3.151 |
| F83 | Retinopathy | T83/82 | Obesity/overweight | 2.003 |
| F92 | Cataract | T83/82 | Obesity/overweight | 1.277 |
| K82 | Pulmonary heart dis. | T83/82 | Obesity/overweight | 1.81 |
| P72 | Schizophrenia | T83/82 | Obesity/overweight | 1.448 |
| T93 | Lipid disorder | T83/82 | Obesity/overweight | 1.402 |
| U88 | Glomerulonephritis/nephrosis | T83/82 | Obesity/overweight | 1.559 |
| D76 | Pancreas Ca. | T89 | Type 1 Diabetes mellitus | 4.01 |
| T90 | Type 2 Diabetes mellitus | T89 | Type 1 Diabetes mellitus | 3.925 |
| B78 | Hereditary haemolytic anaemia | T90 | Type 2 Diabetes mellitus | 1.201 |
| B81 | Anaemia vitamin B12/folate deficiency | T90 | Type 2 Diabetes mellitus | 1.438 |
| B82 | Anaemia other, unspecified | T90 | Type 2 Diabetes mellitus | 1.725 |
| D77 | Digestive ca. other/NOS | T90 | Type 2 Diabetes mellitus | 1.222 |
| D97 | Liver dis. NOS | T90 | Type 2 Diabetes mellitus | 1.89 |
| D98 | Cholecystitis/cholelithiasis | T90 | Type 2 Diabetes mellitus | 1.341 |
| D99 | Dis. digestive system, other | T90 | Type 2 Diabetes mellitus | 1.587 |
| F92 | Cataract | T90 | Type 2 Diabetes mellitus | 1.379 |
| K74 | Ischaemic heart dis. with angina | T90 | Type 2 Diabetes mellitus | 1.894 |
| K76 | Ischaemic heart dis. without angina | T90 | Type 2 Diabetes mellitus | 2.161 |
| K77 | Heart failure | T90 | Type 2 Diabetes mellitus | 1.867 |
| K78 | Atrial Fibrillation/flutter | T90 | Type 2 Diabetes mellitus | 1.372 |
| K86 | Hypertension uncomplicated | T90 | Type 2 Diabetes mellitus | 2.759 |
| K89 | Transient cerebral ischaemia | T90 | Type 2 Diabetes mellitus | 1.246 |
| K90 | Stroke | T90 | Type 2 Diabetes mellitus | 1.644 |
| N93 | Carpal tunnel syndrome | T90 | Type 2 Diabetes mellitus | 1.24 |
| P71 | Psychosis organic, other | T90 | Type 2 Diabetes mellitus | 1.209 |
| P72 | Schizophrenia | T90 | Type 2 Diabetes mellitus | 1.293 |
| T83/82 | Obesity/overweight | T90 | Type 2 Diabetes mellitus | 2.584 |
| T89 | Type 1 Diabetes mellitus | T90 | Type 2 Diabetes mellitus | 3.925 |
| T93 | Lipid disorder | T90 | Type 2 Diabetes mellitus | 1.691 |
| T99 | Endocrine/metabolic/nutrition dis. other | T90 | Type 2 Diabetes mellitus | 1.31 |
| U99.01 | Chronic renal failure | T90 | Type 2 Diabetes mellitus | 1.763 |
| X77 | Female genital ca, other | T90 | Type 2 Diabetes mellitus | 1.38 |
| B82 | Anaemia other, unspecified | T92 | Gout | 1.622 |
| D97 | Liver dis. NOS | T92 | Gout | 1.508 |
| D98 | Cholecystitis/cholelithiasis | T92 | Gout | 1.274 |
| K74 | Ischaemic heart dis. with angina | T92 | Gout | 1.316 |
| K75 | Acute myocardial infarction | T92 | Gout | 1.204 |
| K76 | Ischaemic heart dis. without angina | T92 | Gout | 1.293 |
| K77 | Heart failure | T92 | Gout | 1.922 |
| K78 | Atrial Fibrillation/flutter | T92 | Gout | 1.775 |
| K82 | Pulmonary heart dis. | T92 | Gout | 2.056 |
| K84 | Heart dis. other | T92 | Gout | 1.45 |
| K86 | Hypertension uncomplicated | T92 | Gout | 2.43 |
| K87 | Hypertension complicated | T92 | Gout | 1.818 |
| K89 | Transient cerebral ischaemia | T92 | Gout | 1.243 |
| K92 | Atherosclerosis/peripheral vascular dis | T92 | Gout | 1.278 |
| N93 | Carpal tunnel syndrome | T92 | Gout | 1.319 |
| T83/82 | Obesity/overweight | T92 | Gout | 2.004 |
| T90 | Type 2 Diabetes mellitus | T92 | Gout | 1.346 |
| T93 | Lipid disorder | T92 | Gout | 1.566 |
| T99 | Endocrine/metabolic/nutrition dis. other | T92 | Gout | 5.105 |
| U88 | Glomerulonephritis/nephrosis | T92 | Gout | 1.931 |
| U99.01 | Chronic renal failure | T92 | Gout | 3.254 |
| X77 | Female genital ca, other | T92 | Gout | 1.342 |
| F92 | Cataract | T93 | Lipid disorder | 1.265 |
| F93 | Glaucoma | T93 | Lipid disorder | 1.227 |
| K75 | Acute myocardial infarction | T93 | Lipid disorder | 1.728 |
| K84 | Heart dis. other | T93 | Lipid disorder | 1.253 |
| K86 | Hypertension uncomplicated | T93 | Lipid disorder | 1.686 |
| K87 | Hypertension complicated | T93 | Lipid disorder | 1.444 |
| K89 | Transient cerebral ischaemia | T93 | Lipid disorder | 1.311 |
| K90 | Stroke | T93 | Lipid disorder | 1.204 |
| K91 | Cerebrovascular dis. | T93 | Lipid disorder | 1.459 |
| K92 | Atherosclerosis/peripheral vascular dis | T93 | Lipid disorder | 1.42 |
| T83/82 | Obesity/overweight | T93 | Lipid disorder | 1.402 |
| T90 | Type 2 Diabetes mellitus | T93 | Lipid disorder | 1.691 |
| T99 | Endocrine/metabolic/nutrition dis. other | T93 | Lipid disorder | 1.44 |
| U88 | Glomerulonephritis/nephrosis | T93 | Lipid disorder | 1.52 |
| U99.01 | Chronic renal failure | T93 | Lipid disorder | 1.463 |
| B81 | Anaemia vitamin B12/folate deficiency | T99 | Endocrine/metabolic/nutrition dis. other | 1.362 |
| B82 | Anaemia other, unspecified | T99 | Endocrine/metabolic/nutrition dis. other | 1.599 |
| F84 | Macular degeneration | T99 | Endocrine/metabolic/nutrition dis. other | 1.205 |
| F92 | Cataract | T99 | Endocrine/metabolic/nutrition dis. other | 1.273 |
| K77 | Heart failure | T99 | Endocrine/metabolic/nutrition dis. other | 1.773 |
| K78 | Atrial Fibrillation/flutter | T99 | Endocrine/metabolic/nutrition dis. other | 1.541 |
| K82 | Pulmonary heart dis. | T99 | Endocrine/metabolic/nutrition dis. other | 2.05 |
| K84 | Heart dis. other | T99 | Endocrine/metabolic/nutrition dis. other | 1.49 |
| K86 | Hypertension uncomplicated | T99 | Endocrine/metabolic/nutrition dis. other | 1.985 |
| K87 | Hypertension complicated | T99 | Endocrine/metabolic/nutrition dis. other | 1.967 |
| K91 | Cerebrovascular dis. | T99 | Endocrine/metabolic/nutrition dis. other | 1.201 |
| K92 | Atherosclerosis/peripheral vascular dis | T99 | Endocrine/metabolic/nutrition dis. other | 1.318 |
| T83/82 | Obesity/overweight | T99 | Endocrine/metabolic/nutrition dis. other | 1.679 |
| T90 | Type 2 Diabetes mellitus | T99 | Endocrine/metabolic/nutrition dis. other | 1.31 |
| T93 | Lipid disorder | T99 | Endocrine/metabolic/nutrition dis. other | 1.44 |
| U88 | Glomerulonephritis/nephrosis | T99 | Endocrine/metabolic/nutrition dis. other | 1.929 |
| U99.01 | Chronic renal failure | T99 | Endocrine/metabolic/nutrition dis. other | 3.233 |
| B81 | Anaemia vitamin B12/folate deficiency | U88 | Glomerulonephritis/nephrosis | 1.425 |
| B82 | Anaemia other, unspecified | U88 | Glomerulonephritis/nephrosis | 2.119 |
| D97 | Liver dis. NOS | U88 | Glomerulonephritis/nephrosis | 1.282 |
| D99 | Dis. digestive system, other | U88 | Glomerulonephritis/nephrosis | 1.61 |
| F83 | Retinopathy | U88 | Glomerulonephritis/nephrosis | 5.951 |
| K74 | Ischaemic heart dis. with angina | U88 | Glomerulonephritis/nephrosis | 1.603 |
| K75 | Acute myocardial infarction | U88 | Glomerulonephritis/nephrosis | 1.717 |
| K76 | Ischaemic heart dis. without angina | U88 | Glomerulonephritis/nephrosis | 1.893 |
| K77 | Heart failure | U88 | Glomerulonephritis/nephrosis | 1.727 |
| K78 | Atrial Fibrillation/flutter | U88 | Glomerulonephritis/nephrosis | 1.428 |
| K82 | Pulmonary heart dis. | U88 | Glomerulonephritis/nephrosis | 1.833 |
| K84 | Heart dis. other | U88 | Glomerulonephritis/nephrosis | 1.532 |
| K86 | Hypertension uncomplicated | U88 | Glomerulonephritis/nephrosis | 3.021 |
| K89 | Transient cerebral ischaemia | U88 | Glomerulonephritis/nephrosis | 1.346 |
| K91 | Cerebrovascular dis. | U88 | Glomerulonephritis/nephrosis | 1.625 |
| K92 | Atherosclerosis/peripheral vascular dis | U88 | Glomerulonephritis/nephrosis | 2.188 |
| N94 | Peripheral neuritis/neuropathy | U88 | Glomerulonephritis/nephrosis | 2.713 |
| T83/82 | Obesity/overweight | U88 | Glomerulonephritis/nephrosis | 1.559 |
| T89 | Type 1 Diabetes mellitus | U88 | Glomerulonephritis/nephrosis | 7.333 |
| T90 | Type 2 Diabetes mellitus | U88 | Glomerulonephritis/nephrosis | 3.443 |
| T92 | Gout | U88 | Glomerulonephritis/nephrosis | 1.931 |
| T93 | Lipid disorder | U88 | Glomerulonephritis/nephrosis | 1.52 |
| D99 | Dis. digestive system, other | U99.01 | Chronic renal failure | 1.62 |
| F83 | Retinopathy | U99.01 | Chronic renal failure | 2.695 |
| F84 | Macular degeneration | U99.01 | Chronic renal failure | 1.34 |
| F92 | Cataract | U99.01 | Chronic renal failure | 1.428 |
| K74 | Ischaemic heart dis. with angina | U99.01 | Chronic renal failure | 1.771 |
| K75 | Acute myocardial infarction | U99.01 | Chronic renal failure | 1.932 |
| K77 | Heart failure | U99.01 | Chronic renal failure | 2.561 |
| K82 | Pulmonary heart dis. | U99.01 | Chronic renal failure | 2.569 |
| K84 | Heart dis. other | U99.01 | Chronic renal failure | 1.784 |
| K86 | Hypertension uncomplicated | U99.01 | Chronic renal failure | 3.456 |
| K87 | Hypertension complicated | U99.01 | Chronic renal failure | 2.332 |
| K89 | Transient cerebral ischaemia | U99.01 | Chronic renal failure | 1.402 |
| K90 | Stroke | U99.01 | Chronic renal failure | 1.46 |
| K91 | Cerebrovascular dis. | U99.01 | Chronic renal failure | 1.55 |
| K92 | Atherosclerosis/peripheral vascular dis | U99.01 | Chronic renal failure | 2.094 |
| N94 | Peripheral neuritis/neuropathy | U99.01 | Chronic renal failure | 1.619 |
| P71 | Psychosis organic, other | U99.01 | Chronic renal failure | 1.399 |
| T83/82 | Obesity/overweight | U99.01 | Chronic renal failure | 1.579 |
| T89 | Type 1 Diabetes mellitus | U99.01 | Chronic renal failure | 2.151 |
| T90 | Type 2 Diabetes mellitus | U99.01 | Chronic renal failure | 1.763 |
| T93 | Lipid disorder | U99.01 | Chronic renal failure | 1.463 |
| U88 | Glomerulonephritis/nephrosis | U99.01 | Chronic renal failure | 2.421 |
| F83 | Retinopathy | X77 | Female genital ca, other | 1.26 |
| P72 | Schizophrenia | X77 | Female genital ca, other | 1.42 |
| T83/82 | Obesity/overweight | X77 | Female genital ca, other | 1.319 |
| T92 | Gout | X77 | Female genital ca, other | 1.342 |

**Table S8. Temporal comorbid associations. Conditions receiving immediate connections from different diseases in T2DM trajectories.**

| ICPC2 code | Chronic condition | nº connections from different diseases | Precedent diseases |
| --- | --- | --- | --- |
| K82 | Pulmonary heart dis. | 10 | Retinopathy, Ischaemic heart dis. with angina, Acute myocardial infarction, Ischaemic heart dis. without angina, Heart failure, Atrial Fibrillation/flutter, Heart dis. other, Hypertension complicated, Peripheral neuritis/neuropathy, type 2 diabetes |
| D99 | Dis. digestive system, other | 7 | Retinopathy, Pancreas Ca, Atherosclerosis/peripheral vascular dis, Anaemia vitamin B12/folate deficiency, Endocrine/metabolic/nutrition dis. other, Ischaemic heart dis. without angina, Ischaemic heart dis. with angina |
| K78 | Atrial Fibrillation/flutter | 7 | Acute myocardial infarction, Ischaemic heart dis. without angina, Heart dis. other, Hypertension complicated, Transient cerebral ischaemia, Atherosclerosis/peripheral vascular dis, Chronic renal failure |
| K76 | Ischaemic heart dis. without angina | 6 | Peripheral neuritis/neuropathy, Acute myocardial infarction, Heart dis. other, Chronic renal failure, Stroke, Ischaemic heart dis. with angina |
| K77 | Heart failure | 5 | Retinopathy, Hypertension complicated, Heart dis. other, Ischaemic heart dis. without angina, Ischaemic heart dis. with angina |
| U99.01 | Chronic renal failure | 5 | Retinopathy, Glomerulonephritis/nephrosis, Peripheral neuritis/neuropathy, Stroke, Ischaemic heart dis. with angina |
| K87 | Hypertension complicated | 5 | Retinopathy, Glomerulonephritis/nephrosis, Atherosclerosis/peripheral vascular dis, Stroke, type 2 diabetes |
| N94 | Peripheral neuritis/neuropathy | 5 | Cerebrovascular dis., Anaemia vitamin B12/folate deficiency, Stroke, Ischaemic heart dis. with angina, type 2 diabetes |
| K91 | Cerebrovascular dis. | 4 | Retinopathy, Transient cerebral ischaemia, Stroke, Acute myocardial infarction |
| T99 | Endocrine/metabolic/nutrition dis. other | 4 | Pulmonary heart dis., Hypertension complicated, Glomerulonephritis/nephrosis, Chronic renal failure |
| K84 | Heart dis. other | 4 | Retinopathy, Hypertension complicated, Heart dis. other, Ischaemic heart dis. without angina, Ischaemic heart dis. with angina, Cataract, type 2 diabetes |
| U88 | Glomerulonephritis/nephrosis | 4 | Heart dis. other, Ischaemic heart dis. without angina, Ischaemic heart dis. with angina, type 2 diabetes |
| T92 | Gout | 3 | Chronic renal failure, Endocrine/metabolic/nutrition dis. other, Atrial Fibrillation/flutter |
| F92 | Cataract | 3 | Glomerulonephritis/nephrosis, Peripheral neuritis/neuropathy, Glaucoma |
| K90 | Stroke | 3 | Glomerulonephritis/nephrosis, Heart failure, Atrial Fibrillation/flutter |
| B82 | Anaemia other, unspecified | 3 | Chronic renal failure, Heart failure, Ischaemic heart dis. with angina |
| K75 | Acute myocardial infarction | 3 | Retinopathy, Peripheral neuritis/neuropathy, type 2 diabetes |
| B81 | Anaemia vitamin B12/folate deficiency | 2 | Retinopathy, Chronic renal failure |
| F93 | Glaucoma | 2 | Retinopathy, type 2 diabetes |
| K92 | Atherosclerosis/peripheral vascular dis | 2 | Ischaemic heart dis. without angina, type 2 diabetes |

Trajectories include up to three steps from type 2 diabetes mellitus with OR >1.5

**Table S9. Sensitivity analysis for T2DM networks. Whole population (ICPC-2) versus patients with more complete data (ICPC-2 HQ) for different threshold OR values.**

|  | Number of nodes | | | Number of links | | |
| --- | --- | --- | --- | --- | --- | --- |
| OR | ICPC-2 | ICPC-2 HQ | Difference | ICPC-2 | ICPC2- HQ | Difference (%variation) |
| 1.2 | 38 | 37 | 1 | 439 | 398 | 41 (9%) |
| 1.3 | 31 | 31 | 0 | 306 | 281 | 25 (8%) |
| 1.4 | 25 | 25 | 0 | 204 | 177 | 27 (13%) |
| 1.5 | 23 | 23 | 0 | 156 | 150 | 6 (4%) |
| 1.6 | 21 | 20 | 1 | 125 | 111 | 14 (11%) |
| 1.7 | 16 | 16 | 0 | 72 | 68 | 4 (6%) |
| 1.8 | 14 | 14 | 0 | 47 | 47 | 0 (0%) |
| 1.9 | 11 | 11 | 0 | 32 | 31 | 1 (3%) |
| 2 | 11 | 10 | 1 | 32 | 24 | 8 (25%) |

**Table S10. Sensitivity analysis for T2DM networks. Temporal association criteria: probabilities of <40% or >60% versus <20% or >80%of previous/subsequent diagnosis among pairs of chronic conditions.**

|  | Number of directional interactions | | |
| --- | --- | --- | --- |
| OR | <40% or >60% | <20% or > 80% | Difference (%variation) |
| 1.2 | 254 | 40 | 214 (84%) |
| 1.3 | 206 | 36 | 170 (83%) |
| 1.4 | 175 | 31 | 144 (82%) |
| 1.5 | 146 | 28 | 118 (81%) |
| 1.6 | 120 | 27 | 93 (78%) |
| 1.7 | 99 | 24 | 75 (76%) |
| 1.8 | 85 | 20 | 65 (76%) |
| 1.9 | 71 | 16 | 55 (77%) |
| 2 | 63 | 12 | 51 (81%) |

**Table S11. Directional associations in T2DM directed networks using <20% or >80% criteria**

| From (code) | From (label) | To (code) | To (label) | OR |
| --- | --- | --- | --- | --- |
| T89 | T1DM | F83 | Retinopathy | 24.68 |
| T90 | T2DM | F83 | Retinopathy | 22.84 |
| K75 | Acute myocardial infarction | K76 | Ischaemic heart disease without angina | 12.19 |
| K86 | Hypertension uncomplicated | K87 | Hypertension complicated | 9.10 |
| T89 | T1DM | U88 | Glomerulonephritis/nephrosis | 7.38 |
| T89 | T1DM | N94 | Peripheral neuritis/neuropathy | 5.19 |
| T89 | T1DM | K92 | Atherosclerosis/PVD | 3.13 |
| K77 | Heart failure | U99.01 | Chronic renal failure | 2.61 |
| U99.01 | Chronic renal failure | K77 | Heart failure | 2.61 |
| T89 | T1DM | U99.01 | Chronic renal failure | 2.23 |
| F83 | Retinopathy | K75 | Acute myocardial infarction | 2.12 |
| K92 | Atherosclerosis/PVD | D99 | Disease digestive system, other | 2.04 |
| T89 | T1DM | F93 | Glaucoma | 1.93 |
| F83 | Retinopathy | K74 | Ischaemic heart disease with angina | 1.91 |
| K74 | Ischaemic heart disease with angina | F83 | Retinopathy | 1.91 |
| T89 | T1DM | K90 | Stroke | 1.90 |
| K74 | Ischaemic heart disease with angina | T90 | T2DM | 1.87 |
| K87 | Hypertension complicated | T83/82 | Overweight/Obesity | 1.86 |
| T83/82 | Overweight/Obesity | K87 | Hypertension complicated | 1.86 |
| K86 | Hypertension uncomplicated | K74 | Ischaemic heart disease with angina | 1.81 |
| F83 | Retinopathy | T83/82 | Overweight/Obesity | 1.77 |
| T83/82 | Overweight/Obesity | F83 | Retinopathy | 1.77 |
| K86 | Hypertension uncomplicated | K76 | Ischaemic heart disease without angina | 1.73 |
| T93 | Lipid disorder | K76 | Ischaemic heart disease without angina | 1.73 |
| T93 | Lipid disorder | K74 | Ischaemic heart disease with angina | 1.69 |
| F83 | Retinopathy | D99 | Disease digestive system, other | 1.62 |
| T90 | T2DM | K87 | Hypertension complicated | 1.61 |
| N94 | Peripheral neuritis/neuropathy | U99.01 | Chronic renal failure | 1.53 |
| K86 | Hypertension uncomplicated | K75 | Acute myocardial infarction | 1.44 |
| T89 | T1DM | B82 | Anaemia other/unspecified | 1.43 |
| T89 | T1DM | N93 | Carpal tunnel syndrome | 1.42 |
| F83 | Retinopathy | P71 | Organic psychosis other | 1.39 |
| F92 | Cataract | K92 | Atherosclerosis/PVD | 1.36 |
| P72 | Schizophrenia | T90 | T2DM | 1.33 |
| F92 | Cataract | P71 | Organic psychosis other | 1.31 |
| T89 | T1DM | K86 | Hypertension uncomplicated | 1.31 |
| N93 | Carpal tunnel syndrome | T92 | Gout | 1.27 |
| T90 | T2DM | F84 | Macular degeneration | 1.27 |
| T99 | Endocrine/metabolic/nutrition disease, other | K76 | Ischaemic heart disease without angina | 1.27 |
| K76 | Ischaemic heart disease without angina | P71 | Organic psychosis other | 1.24 |
